# 12-months metabolic changes among gender dysphoric individuals under cross-sex hormone treatment: a targeted metabolomics study

**DOI:** 10.1038/srep37005

**Published:** 2016-11-11

**Authors:** Matthias K. Auer, Alexander Cecil, Yasmin Roepke, Charlotte Bultynck, Charlotte Pas, Johannes Fuss, Cornelia Prehn, Rui Wang-Sattler, Jerzy Adamski, Günter K. Stalla, Guy T’Sjoen

**Affiliations:** 1 Endocrinology, Diabetology and Internal Medicine, Max Planck Institute of Psychiatry, Munich, Germany; 2 Helmholtz Zentrum München, German Research Center for Environmental Health, Institute of Experimental Genetics, Genome Analysis Center, Neuherberg, Germany; 3 Department of Endocrinology and Center for Sexology and Gender, Ghent University Hospital, Ghent, Belgium; 4 Institute for Sex Research and Forensic Psychiatry, University Medical Center Hamburg-Eppendorf, Hamburg, Germany; 5 Institute of Epidemiology II, Helmholtz Zentrum München, Neuherberg, Germany; 6 Lehrstuhl für Experimentelle Genetik, Technische Universität München, Freising-Weihenstephan, Germany; 7 German Center for Diabetes Research (DZD), Neuherberg, Germany

## Abstract

Metabolomic analyses in epidemiological studies have demonstrated a strong sexual dimorphism for most metabolites. Cross-sex hormone treatment (CSH) in transgender individuals enables the study of metabolites in a cross-gender setting. Targeted metabolomic profiling of serum of fasting transmen and transwomen at baseline and following 12 months of CSH (N = 20/group) was performed. Changes in 186 serum metabolites and metabolite ratios were determined by targeted metabolomics analysis based on ESI-LC-MS/MS. RandomForest (RF) analysis was applied to detect metabolites of highest interest for grouping of transwomen and transmen before and after initiation of CSH. Principal component analysis (PCA) was performed to check whether group differentiation was achievable according to these variables and to see if changes in metabolite levels could be explained by a priori gender differences. PCA predicted grouping of individuals-determined by the citrulline/arginine-ratio and the amino acids lysine, alanine and asymmetric dimethylarginine - in addition to the expected grouping due to changes in sex steroids and body composition. The fact that most of the investigated metabolites did, however, not change, indicates that the majority of sex dependent differences in metabolites reported in the literature before may primarily not be attributable to sex hormones but to other gender-differences.

Transgender individuals are characterized by incongruence between gender identity and external sexual anatomy at birth. An etiological reason for this phenomenon has not been identified so far, but psychological and biological factors have been discussed in this context^1^,^2^,^3^,^4^. To mitigate the feeling of gender dysphoria, interventions such as cross-sex hormone treatment (CSH) and gender affirming surgeries (GAS) are applied in medical care of transgender persons.

Many diseases, such as autoimmune^5^, psychiatric^6^ or cardiovascular diseases^7^, show a sexual dimorphism with respect to prevalence rates. The underlying reasons are not completely understood but it is anticipated that there are multifactorial explanations for these findings^5^,^6^,^7^,^8^. In addition to the different genetic and psychosocial background of men and women^7^, sex steroids are suggested to have a significant effect on these outcomes^9^,^10^,^11^. CSH in transgender persons enables, to some extent, the study of the role of sex steroids in disease pathophysiology partially uncoupled from other sex-specific influential factors.

With regard to metabolism, it is already known that CSH results in impressive changes in the physical appearance towards the target-sex^12^,^13^ as well as in classical cardiovascular risk factors, such as the HDL-/LDL cholesterol ratio, triglycerides^12^, fasting insulin levels, blood pressure^14^ or arterial stiffness^15^.

One technique that may shed light on so far unrevealed metabolic changes following the initiation of CSH is metabolomics. Metabolomics analyzes small molecules (or metabolites) of 80–1200 Da molecular mass in a biological sample^16^. The metabolome represents all metabolites regarded as the ultimate product resulting from the cascade of gene, mRNA and protein expression. Metabolites are considered as close proxies to the corresponding phenotype^17^. Although sex hormones are known to exert a variety of effects on metabolism^18^, this technique has rarely been used in this context^19^,^20^ so far, though it might provide a lot more information on altered metabolic processes than those provided by conventional laboratory analyses.

Interestingly, it has been demonstrated in epidemiological samples that the majority of metabolites measured seem to be differentially regulated between men and women^21^. In a large population-based epidemiological study with more than 3000 participants, more than 101 out of the 131 investigated metabolites presented a sex-dependent dimorphism^22^. To what extent these differences can be attributed to a genetic basis or to the different sex hormonal milieu has not yet been investigated. Applying this technique to a sample of transgender persons before and after initiation of CSH treatment may hence shed light on the general sex-hormone-accountable effect of sex-specific metabolite levels. In the following, we are therefore reporting the results of a prospective study in transgender persons investigating the effects of the initiation of CSH treatment by applying a targeted-metabolomics approach.

## Materials and Methods

### Patient population

Subjects investigated in this study were part of the European Network for the Investigation of Gender Incongruence (ENIGI), a collaboration of four European gender identity clinics (Amsterdam, Ghent, Hamburg, and Oslo) for the study of the diagnostics and treatment of transsexualism^12^.

All participants in this study had been diagnosed and treated at the Department of Endocrinology at Ghent University Hospital between February 2010 and August 2012. In total N = 20 Caucasian individuals per group were investigated at baseline and following 12 months of CSH treatment. All transwomen (MtF) were hormone naïve at baseline, while N = 6 transmen (FtM) had already received 5 mg Lynestrenol/d (Orgametril) in order to stop their menstrual cycle. All FtM were put on cross-sex hormone treatment with 1000 mg Testosterone (T) –undecanoate (Nebido, Jenapharm) every three months. The most appropriate mode of hormone treatment in MtF was chosen depending on the age of the corresponding subject, in view of the increased cardiovascular risk profile in older MtF. MtF below the age of 45 years (N = 13) were treated with 50 mg of Cyproteronacetat (CA) (Androcur, Bayer) in addition to 4 mg of estradiol valerate (EV) (Progynova, Bayer), both given orally once a day, while those being older than 45 years received 50 mg of CA daily and a transdermal 17-β-estradiol (E2) patch releasing 100 μg/24 hours (Dermestril, Besins, Belgium) (N = 7).

This study was approved by the ethical review board of the Ghent University Hospital and the University Hospital of Oslo. The study was conducted in accordance with the Declaration of Helsinki and all participants gave written informed consent. This study is registered at clinical trials.gov. Clinical trial number: NCT01072825.

### Medical History and Examination

Data on comorbidities and life-style, including smoking history, alcohol intake, medical history and medication use, were acquired from each patient by means of questionnaires and corresponding information was compared to the clinical information from the clinical data collected in the medical chart files. Physical activity was measured using the Baecke habitual physical activity questionnaire^23^.

### Anthropometrics and laboratory measures

Assessments of anthropometry and laboratory measurements have already been described elsewhere^12^. Briefly, height was measured to the nearest 0.1 cm using a Harpendenstadiometer (HoltainLtd, Crymuch, UK) and body weight was measured in light indoor clothing without shoes to the nearest 0.5 kg. Body composition was assessed by means of dual-energy X ray absorptiometry with a Hologic Discovery Machine (Hologic Inc., Bedford, MA, USA). Serum samples were taken in the morning between 8:00 and 9:00 a.m. following an overnight fast. After a clotting period of 30–60 minutes, serum was immediately centrifuged and stored at −80 °C until further analysis. 17-β-Estradiol (E2) and testosterone were determined using liquid chromatography tandem mass spectrometry (AB Sciex 5,500 triplequadrupole mass spectrometer; AB Sciex, Toronto, Canada). Immunoassays were used to determine follicle-stimulating hormone (FSH), luteinizing hormone (LH), leptin and sex hormone binding globulin (SHBG) and interassay-coefficients of variance (CVs), please see ref. 12.

### Targeted metabolomics

The targeted metabolomics approach was based on ESI-LC-MS/MS measurements by Absolute*IDQ* p180 Kit (BIOCRATES Life Sciences AG, Innsbruck, Austria). The assay allows simultaneous quantification of 188 metabolites out of 10 μL plasma and includes free carnitine, 39 acylcarnitines (Cx:y), 21 amino acids (19 proteinogenic + citrulline + ornithine), 21 biogenic amines, hexoses (sum of hexoses – about 90–95% glucose), 90 glycerophospholipids (14 lysophosphatidylcholines (lysoPC) and 76 phosphatidylcholines (PC)) and 15 sphingolipids (SMx:y). The abbreviations Cx:y are used to describe the total number of carbons and double bonds of all chains, respectively (for more details, see ref. 24). The method of Absolute*IDQ* p180 Kit has been proven to be in conformance with FDA-Guideline “Guidance for Industry-Bioanalytical Method Validation^25^, which implies proof of reproducibility within a given error range. Measurements were performed as described in the manufacturer in manual UM-P180. The LLOQ and ULOQ were determined experimentally by Biocrates.

Assay procedures of the Absolute*IDQ* p180 Kit have been described in detail previously^24^,^26^. Metabolite nomenclature can be found in the supplements (Supplementary Table 1).

Sample handling was performed by a Hamilton Microlab STAR robot (Hamilton Bonaduz AG, Bonaduz, Switzerland) and an Ultravap nitrogen evaporator (Porvair Sciences, Leatherhead, U.K.), in addition to standard laboratory equipment. Mass spectrometric (MS) analyses were done on an API 4000 LC/MS/MS System (Sciex Deutschland GmbH, Darmstadt, Germany) equipped with a 1200 Series HPLC (Agilent Technologies Deutschland GmbH, Böblingen, Germany) and a HTC PAL auto sampler (CTC Analytics, Zwingen, Switzerland) controlled by the software Analyst 1.5.1. Data evaluation for quantification of metabolite concentrations and quality assessment was performed with the Met*IDQ* software package, an integral part of the Absolute*IDQ* Kit. Internal standards served as reference for the calculation of metabolite concentrations [μM].

### Statistical analysis

Data were analyzed using R software, v.3.1.2. Descriptive statistics were expressed as means and standard deviations. Statistical analyses of categorical variables were carried out using the χ2-test. Statistics of means in prospective data were carried out using the paired student t-test for continuous variables. QQ plots were used to assess normality of distribution. McNemar’s test was used for dichotomous variables. Significance was set at the p < 0.05 level (two-tailed). To select the most interesting metabolites for Principal Component Analysis (PCA) plotting, a randomforest (RF) analysis was performed^27^ in R^28^.

## Results

### General characteristics

#### Transwomen (MtF)

Twelve months of CSH resulted in significant changes in terms of body composition. While there was no change in anthropometric measures, such as weight or BMI, CSH led to a significant decrease in the percentage of lean body mass and corresponding increase in relative fat mass (p < 0.001). Induction of hormonal therapy did not seem to significantly change any life-style variables, including smoking behavior, physical activity or alcohol intake. As intended, there were several significant changes in terms of hormone measurements. There was a significant increase in serum estradiol levels and a significant decrease in total and free testosterone levels (p < 0.001). As a result, hematocrit levels were decreased after 12 months of continuous CSH (p = 0.002). LH and FSH were significantly suppressed at follow-up (p < 0.001). CSH further resulted in a significant increase in leptin (p < 0.001) and insulin levels (p = 0.018). This caused a significant increase in HOMA index as a measure of insulin resistance (p = 0.040), while fasting glucose was not affected.

CSH further led to a significant increase in HDL levels (p = 0.039) but no changes in LDL or total cholesterol levels.

At baseline, those who received transdermal E2 in contrast to those receiving oral E2 only differed in terms of physical activity (p = 0.002) and free testosterone (p = 0.023), in accordance with the higher age of this subgroup. At follow-up, there was only a persisting difference in terms of physical activity in general and sport activity in particular (p = 0.036) between oral and transdermal E2 users. In particular, individuals did not differ in terms of other sex hormone levels or anthropometric measures (data not shown). Thus, they were regarded as only one group (Table 1) in the following analysis.

#### Transmen (FtM)

Testosterone treatment did not affect BMI or weight but significantly increased lean mass (p = 0.030) in transmen. Total and free testosterone (T) levels were significantly higher at follow-up and estradiol levels significantly lower (p < 0.001). SHBG (p = 0.009) and leptin (p = 0.003) were both lower at follow-up. Any changes in terms of life-style variables were not observed. Fasting glucose as well as insulin levels remained unaltered as did lipid levels.

At baseline, there were significant differences in terms of BMI (p = 0.018) and weight (p = 0.020) between those with preceding progestin intake and those without, while there was no significant difference in terms of lean or total fat mass (Table 2). While E2 (p = 0.036) and SHBG (p = 0.007) levels were lower in those with Lynestrenol pre-treatment, no significant differences could be observed regarding T and LH/FSH-levels. Leptin and insulin levels as well as lifestyle aspects remained stable.

At 12 months’ follow-up, FtM with Lynestrenol pretreatment only differed from those without in terms of lower total testosterone (p < 0.038) and lower SHBG levels (p = 0.009) but not in terms of free T (data not shown).

### Metabolomics

A Chi square test was performed for the whole data set, the combination of physiological patient data and metabolite measurements. Results indicated that measured values and clinical data were not independent from one another.

#### Multivariate analysis and feature selection

**Figure 1:**
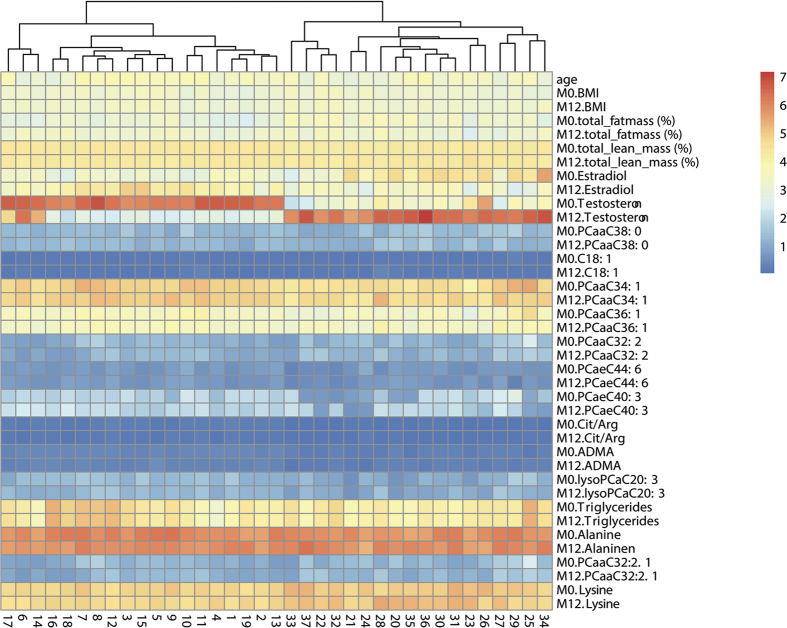
Differentiation of patients by sex hormones, body composition and metabolites. Heatmap analysis shows strong influence of sex hormones administered by CSH. Minute changes in metabolite compositions are masked by the bigger effects of sex hormones involved in CSH. The metabolites shown here were selected by randomForest analysis. *Cx:y: Acylcarnitines* (*The abbreviations Cx:y are used to describe the total number of carbons and double bonds of all chains*). *lysoPC: Lysophosphatidylcholines*. *PC: Phosphatidylcholines SMx:y: Sphingolipids M0: Baseline. M12: After 12 month of cross-sex hormone treatment*.

**Figure 2:**
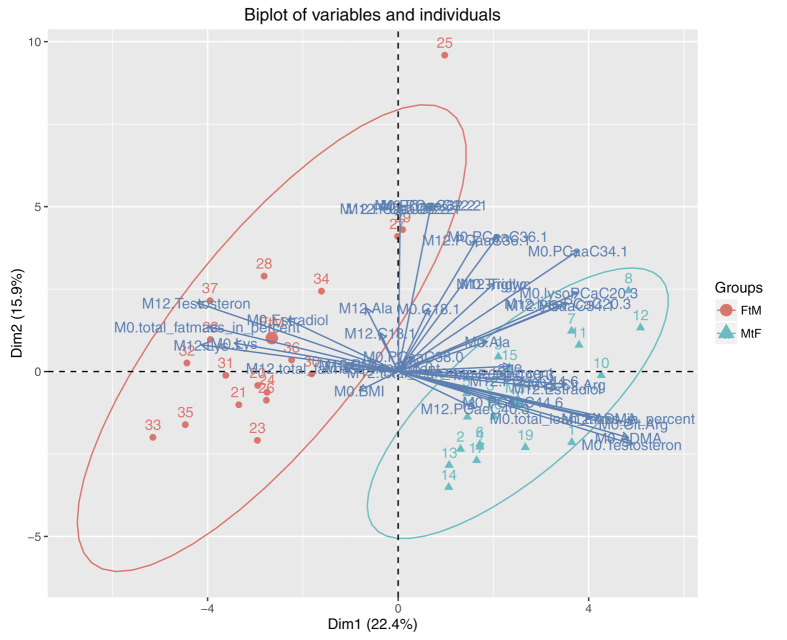
Group clustering by metabolites. This variable factor map of the most important metabolites, as selected by randomForest analysis, was drawn for the differentiation of MtF and FtM patient grouping. The grouping was calculated by PCA. *Cx:y: Acylcarnitines* (*The abbreviations Cx:y are used to describe the total number of carbons and double bonds of all chains*) *lysoPC: Lysophosphatidylcholines. PC: Phosphatidylcholines*. *SMx:y: Sphingolipids*. *M0: Baseline*. *M12: After 12 month of cross-sex hormone treatment*.

RF was applied to detect metabolites with the highest influence for grouping of MtF and FtM patients. Results were then plotted as heatmaps (Fig. 1). Three patients were excluded from analysis due to insufficient data on body composition or sex steroid levels. As there was no clear division into groups possible at this level, principal component analysis (PCA) was performed to check whether differentiation after RF was achievable (Fig. 2). This PCA predicted grouping of individuals highly depends on asymmetric dimethylarginine (ADMA), testosterone, estradiol, the citrulline/arginine-ratio, lysine and body composition. The outliers from the grouping were also most likely defined by metabolites in the northern area of the plot.

**Figure 3:**
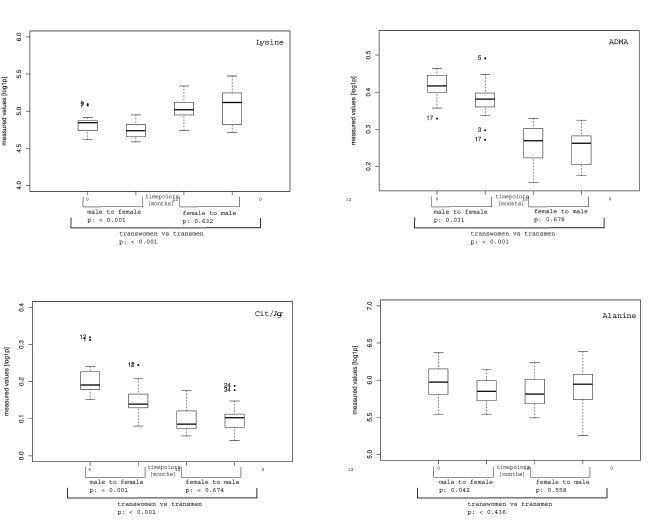
Changes of absolute metabolite concentrations in FtM and MtF following 12 months of CSH. The metabolites shown here were identified by randomForest as well as by PCA.

**Figure 4:**
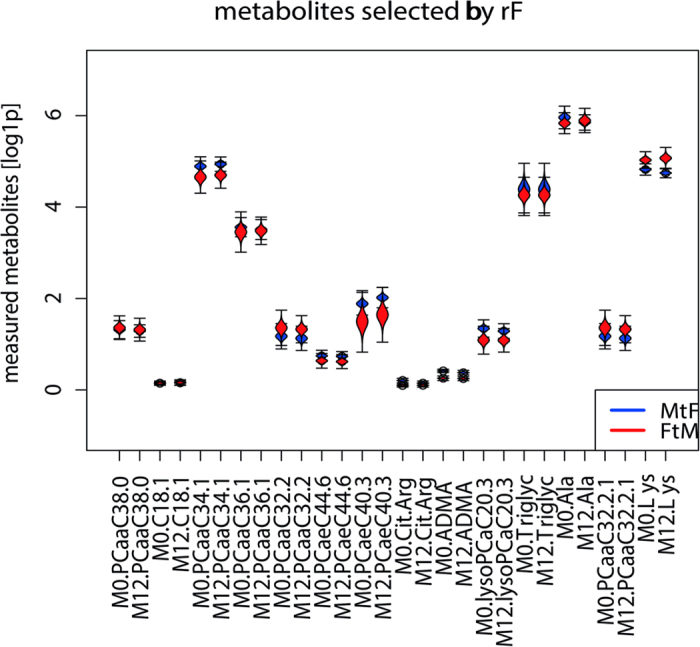
Absolute concentration changes of measured metabolites in FtM and MtF at beginning (0) and after 12 months of survey. *Cx:y: Acylcarnitines (The abbreviations Cx:y are used to describe the total number of carbons and double bonds of all chains)*. *lysoPC: Lysophosphatidylcholines*. *PC: Phosphatidylcholines*. *SMx:y: Sphingolipids*. *M0: Baseline*. *M12: After 12 month of cross-sex hormone treatment.*

By studying the variables identified by RF in detail, a decrease in lysine in FtM was observed, while the opposite was true for MtF. In contrast, there was an increase in alanine in FtM but a decrease in MtF. Following 12 months of CSH, ADMA levels declined in FtM and remained stable in MtF. The strongest effect of CSH was observed for the citrulline/arginine-ratio for both sexes. The citrulline/arginine-ratio was increased in FtM and strongly decreased in MtF (Fig. 3a–d). While some metabolites tended to approach metabolite levels of the target sex in the course of treatment, the concentrations of metabolites remained stable in most cases (Fig. 4).

## Discussion

We found that CSH over 12 months induced a variety of changes in metabolic pathways. As we were particularly interested in variables explaining a priori gender differences, we will focus the following discussion on metabolites having been selected for group separation by RF.

### Metabolism of amino acids

We observed a decrease in alanine and lysine levels in transwomen and an increase in transmen. Free amino acid concentrations (AA) seem to be lower in women than in men in general^29^, and these findings are largely independent of anthropometric measures. In line with this, Burck *et al*. observed a decrease in several free AA in transwomen following CSH and an increase in transmen^30^. The interpretation of this finding is complex, as is AA metabolism.

Concentrations of free AA usually show relatively little inter- or intra-individual variation^31^,^32^ and are primarily determined by the balance of release from endogenous protein stores and utilization by various tissues. In the fasted state, protein breakdown usually exceeds protein synthesis^33^ and free AA primarily reflects release rates from the splanchnic organs.

Alanine is the most abundant free AA^32^. Sources of alanine include the muscle, the gut and, to a lesser extent, the kidneys. Muscle mass accounts for 50% of the total body pool of free AA^31^. It is therefore tempting to ascribe the decrease in alanine and lysine in transwomen to the accompanying decrease in muscle mass. However, these AA have been identified as free fat mass-independent contributors for gender differences in our sample. Sex hormones, especially testosterone, are known regulators of whole body and muscle protein turn-over^34^. Testosterone administration in men results in an increased protein synthesis and reutilization of intracellular AA in skeletal muscles, but not in changes in AA in- or efflux^35^. In line with this, Brodsky and colleagues did show that free AA do not significantly change following six months of testosterone supplementation in hypogonadal men despite an increase in muscle mass and protein synthesis^36^. So far, given the provided background, it may be justified to speculate that the observed AA changes in our sample are primarily determined by direct effects of sex hormones on AA release from the splanchnic region.

### Arginine-Metabolism

In addition, a decrease in the citrulline/arginine-ratio in transwomen and an increase in transmen was observed. This finding was primarily driven by changes in citrulline, while there were no changes in arginine levels. In agreement with this Burk *et al*.^30^ observed a significant decrease in citrulline in transwomen and an increase in transmen, following CSH. The sex steroid dependency of these AA has also been demonstrated in samples of postmenopausal women receiving estrogen replacement therapy resulting in an increase in the citrulline/arginine ratio^37^,^38^. Arginine and citrulline are involved in various metabolic cycles, such as the urea cycle, polyamine metabolism and nitric oxide (NO) synthesis^32^.

**Figure 5:**
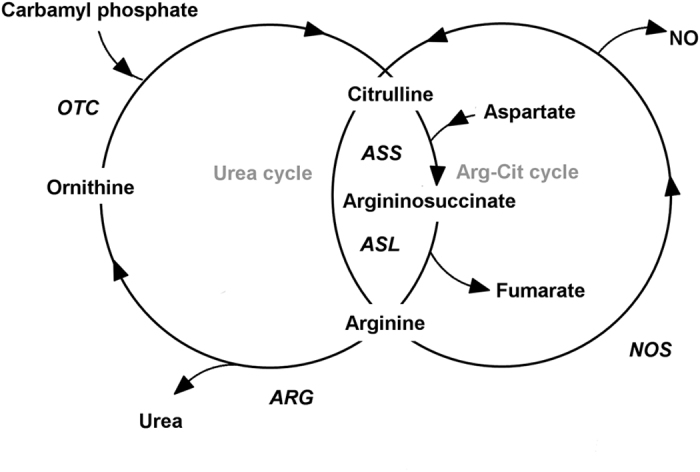
Arginine metabolism. *OTC: Ornithin-Transcarbamylase NO: Nitric oxide NOS: Nitric oxide synthase, ARG: Arginase, ASL: Argininosuccinate-Lyase, ASS: Argininosuccinate synthetase* Table 1.

As a main function, arginine provides the guanido group in the urea cycle, where it is transformed into ornithine during cleavage of urea and finally into citrulline via the reaction with carbamoyl-P^39^ (Fig. 5). Arginine and citrulline are further connected by the arginine-citrulline-cycle, resulting in NO formation^39^. All enzymes necessary to perform the entire urea cycle are exclusively expressed in hepatocytes, while NO synthases (NOS) in contrast are found in various other tissues^40^. The primary sources of NO are endothelial cells^41^, where NO is produced by endothelial NOS (eNOS). As L-Citrulline is the stoichiometric metabolite of NO, the citrulline/arginine-ratio has been suggested as a surrogate marker to estimate NOS activity *in vivo*^42^. This is of particular interest, as diminished eNOS activity is involved in arteriosclerosis development^43^. Although our findings might therefore point to an increase in NOS activity in transmen and a decrease in transwomen, it has to be kept in mind that there is a debate about the reliability and usefulness of the citrulline/arginine-ratio for assessing whole body NOS-activity. This is due to the involvement of these AA in different metabolic pathways and due to the fact that only a small amount of plasma arginine turnover, despite its great physiological relevance, actually seems to be involved in NO-synthesis^44^. This is especially true in comparison to the amounts of these AA being involved in the urea cycle^39^. In addition, the finding that there was also a decrease in the ornithine/arginine-ratio, driven by a decrease in ornithine levels in transwomen, indicates a decrease in arginase activity which transforms arginine into ornithine. In summary, these findings favor the explanation that urea-cycle activity, such as a reduced activity in arginase activity, is responsible for treatment–related alterations in our sample (Fig. 5).

### ADMA

Furthermore, a significant decrease in asymmetric dimethylarginine (ADMA) in transwomen but no corresponding change in transmen was found. ADMA is a methylated derivate of L-arginine. It is generated by activity of protein-methyl-transferase enzymes (PRMTs) methylating protein bound arginine. ADMA is detectable in human blood after being subsequently released into the circulation by proteolysis^45^.

ADMA is a competitive NO synthase inhibitor^45^ and it has further been demonstrated that an increased ADMA/arginine/-ratio can be a risk marker for arteriosclerosis development^46^. In the present study, changes in ADMA in transwomen are in contrast with changes in the citrulline/arginine-ratio, provided that they are a consequence of altered NOS activity. The physiological significance of ADMA as NOS-inhibitor has, however, been questioned, as ADMA concentrations under normal circumstances are very low in comparison to those of arginine^47^.

There is evidence of a role played by sex steroids in ADMA regulation. Females in general seem to have lower ADMA levels than males^48^ and estrogen replacement and progestin administration in postmenopausal women is followed by ADMA decline^49^,^50^,^51^. Studies have further reported a decrease in ADMA levels in middle aged^51^ and younger hypogonadal men^52^, increasing again upon the administration of testosterone replacement therapy^52^. CSH therefore seems to have similar effects in transwomen as estrogen replacement in cissexual women and similar effects in transmen as testosterone replacement in cissexual men.

There are some limitations in the present study that have to be kept in mind. First, only 14 individuals in the transmen group were hormone-naïve at baseline, while the other six had already received Lynestrenol at this time point for achieving cessation of the menstrual cycle, which might have influenced metabolic changes as well. This was due to the fact that ENIGI is an observational study. Therefore, our influence as regards control of treatment modalities was limited to a certain extent. The same applies to the potentially intrinsic effects of CPA co-medication in transwomen. In addition, we did not control the cycle phase in the FtM group, which might have further compromised the detection of clear hormonal effects. Finally, we cannot exclude the possibility that the route of estradiol administration in transwomen might have had an effect on metabolite levels itself. Future studies in larger samples should account for such differences.

In conclusion, we are able to show that although CSH brought about several changes in metabolite levels in transgender persons, the majority of a priori gender differences persisted throughout treatment. Even though the sample sizes of our study were relatively small, our data therefore clearly lead to the conclusion that the abundance of reported sex dependent differences in metabolites, under the application of a similar technical setting, is not primarily attributable to the sex hormonal milieu but due to other gender-differences.

## Additional Information

**How to cite this article**: Auer, M. K. *et al*. 12-months metabolic changes among gender dysphoric individuals under cross-sex hormone treatment: a targeted metabolomics study. *Sci. Rep.* **6**, 37005; doi: 10.1038/srep37005 (2016).

**Publisher’s note:** Springer Nature remains neutral with regard to jurisdictional claims in published maps and institutional affiliations.

## Supplementary Material

Supplementary Information

## Figures and Tables

**Table 1:** General characteristics of transwomen.

Antropometry	Mean (SD)		p-value
	Baseline	12 months follow-up	
BMI (kg/m^2^)	23.90 (4.34)	24.28 (4.01)	0.78
Weight (kg)	76.87 (15.45)	78.50 (14.68)	0.743
WTH-Ratio	0.89 (0.06)	0.86 (0.078)	0.11
Total lean mass (kg)	59.49 (10.65)	56.45 (10.02)	0.37
Total fat (%)	18.56 (4.46)	23.92 (3.64)	**<0.001**
Total lean mass (%)	78.1 (4.17)	72.71 (3.45)	**<0.001**
Total fat mass (kg)	14.65 (5.76)	18.86 (5.36)	**0.026**
Grip-strength (kg)	41.71 (7.77)	41.88 (7.03)	0.948
Systolic blood pressure (mmHg)	127.44 (22.65)	123.06 (12.60)	0.482
Diastolic blood pressure (mmHg)	76.94 (13.12)	75.00 (8.83)	0.609
Life style			
Overall physical activity	8.17 (1.63)	8.36 (1.88)	0.736
Current smoking	0.16 (0.37)	0.16 (0.37)	1
Alcohol intake/week (glasses)	2.32 (2.93)	1.95 (3.19)	0.713
*Lab measurements*			
Hormones			
Estradiol (pg/ml	24.36 (7.11)	64.75 (32.93)	**<0.001**
Total testosterone (ng/dl)	591.28 (162.67)	57.65 (131.78)	**<0.001**
Free testosterone (nmol/l)	11.36 (3.46)	1.24 (2.98)	**<0.001**
FSH (μU/ml)	5.50 (4.14)	0.81 (1.44)	**<0.001**
LH (μU/ml)	5.58 (2.34)	0.75 (1.63)	**<0.001**
SHBG (nmol/l)	39.07 (18.14)	44.40 (21.93)	0.425
Leptin (μg/l)	4.89 (4.14)	11.41 (5.24)	**<0.001**
Other			
Hematocrit (%)	45.21 (2.67)	42.73 (1.79)	**0.002**
Total cholesterol (mg/dl)	185.47 (37.43)	166.26 (31.07)	0.094
HDL (mg/dl)	53.95 (11.66)	56.84 (8.50)	**0.039**
LDL (mg/dl)	112.99 (34.16)	103.10 (28.43)	0.339
Triglycerides (mg/dl)	92.32 (52.66)	81.21 (27.47)	0.422
Insulin (mIU/l)	7.67 (3.47)	12.84 (7.86)	**0.018**
Fasting glucose (mg/dl)	87.63 (9.88)	87.06 (13.97)	0.886
HOMA-Index	1.59 (0.87)	2.66 (1.85)	**0.04**

BMI: body mass index; WTH: waist-to-hip; FSH: follicle-stimulating hormone; LH luteinizing hormone HOMA: Homeostasis Model Assessment; HDL: high-density lipoprotein cholesterol LDL: low-density lipoprotein cholesterol.

**Table 2:** General characteristics of transmen.

Antropometry	Mean (SD)		p-value
	Baseline	12 months follow-up	
BMI (kg/m^2^)	23.49 (4.55)	24.87 (3.65)	0.314
Weight (kg)	64.97 (14.19)	67.19 (12.45)	0.605
WTH-Ratio	0.80 (0.09)	0.80 (0.087)	0.905
Total lean mass (kg)	43.34 (7.24)	48.34 (6.21)	**0.03**
Total fat (%)	27.22 (6.98)	24.06 (6.13)	0.151
Total lean mass (%)	69.36 (6.68)	72.69 (5.87)	0.115
Total fat mass (kg)	17.90 (8.08)	16.63 (6.42)	0.6
Grip-strength (kg)	30.21 (7.25)	32.40 (6.09)	0.322
Systolic blood pressure (mmHg)	115.80 (13.64)	117.06 (14.90)	0.789
Diastolic blood pressure (mmHg)	74.80 (11.33)	45.94 (10.10)	0.967
Life style			
Overall physical activity	9.11 (2.25)	9.44 (2.72)	0.685
Current smoking	0.30 (0.47)	0.20 (0.41)	0.478
Alcohol intake/week (glasses)	0.55 (1.43)	1.55 (2.65)	0.147
*Lab measurements*			
Hormones			
Estradiol (pg/ml)	80.69 (57.72)	27.36 (10.62)	**<0.001**
Total testosterone (ng/dl)	42.37 (55.24)	644.04 (253.08)	**<0.001**
Free testosterone (nmol/l)	0.77 (0.90)	14.98 (5.91)	**<0.001**
FSH (μU/ml)	4.44 (2.13)	17.61 (31.36)	0.076
LH (μU/ml)	6.47 (6.39)	14.66 (27.19)	0.205
SHBG (nmol/l)	57.68 (36.10)	33.19 (13.84)	**0.009**
Leptin (μg/l)	15.27 (10.63)	6.63 (4.53)	**0.003**
Other			
Hematocrit (%)	41.33 (2.93)	44.95 (3.24)	**<0.001**
Total cholesterol (mg/dl)	175.3 (31.79)	186.21 (37.38)	0.334
HDL (mg/dl)	53.10 (13.90)	50.16 (10.10)	0.453
LDL (mg/dl)	106.52 (30.39)	116.51 (32.32)	0.453
Triglycerides (mg/dl)	77.95 (41.49)	102.32 (61.02)	0.157
Insulin (mIU/l)	10.27 (6.64)	12.12 (9.60)	0.482
Fasting Glucose (mg/dl)	80.50 (7.67)	80.25 (11.37)	0.936
HOMA-Index	2.08 (1.43)	2.50 (2.34)	0.495

BMI: body mass index; WTH: waist-to-hip; FSH: follicle-stimulating hormone; LH luteinizing hormone HOMA: Homeostasis Model Assessment; HDL: high-density lipoprotein cholesterol LDL: low-density lipoprotein cholesterol.
