# Supplementary material for: 12-months metabolic changes among gender dysphoric individuals under cross-sex hormone treatment: a targeted metabolomics study
Source: Sci Rep. 2016 Nov 11;6:37005. doi: 10.1038/srep37005 (PMC5105120; doi:10.1038/srep37005)
Supplement: Supplementary Information [file srep37005-s1.pdf]

12-months metabolic changes among gender dysphoric individuals under cross-sex hormone treatment: a targeted metabolomics study

Matthias K. Auer[1+*], Alexander Cecil[2*], Yasmin Roepke[1], Charlotte Bultynck[3], Charlotte Pas[3], Johannes Fuss[4], Cornelia Prehn[2], Rui Wang-Sattler[5], Jerzy Adamski[2, 6, 7], Günter K. Stalla[1], Guy T'Sjoen[3]

[1]Endocrinology, Diabetology and Internal Medicine, Max Planck Institute of Psychiatry, Munich, Germany

[2] Helmholtz Zentrum München, German Research Center for Environmental Health, Institute of Experimental Genetics, Genome Analysis Center, Neuherberg, Germany

[3]Department of Endocrinology and Center for Sexology and Gender, Ghent University Hospital, Ghent, Belgium

[4]Institute for Sex Research and Forensic Psychiatry, University Medical Center Hamburg-Eppendorf, Hamburg, Germany

[5]Institute of Epidemiology II, Helmholtz Zentrum München, Neuherberg, Germany

[6] Lehrstuhl für Experimentelle Genetik, Technische Universität München, Freising-Weihenstephan, Germany

[7] German Center for Diabetes Research (DZD), Neuherberg, Germany

Supplement table 1

Metabolite	Commonly used name	Attached Synonyms	Attached HMDB_ID	Attached KEGG_ID	Class
Ac-Orn	Acetylornithine	(2S)-2-acetamido-5-a	HMDB03357	C00437	Amino Acids and Derivatives
ADMA	Asymmetric<br>dimethylarginine<br>Asymmetric<br>dimethylarginine	dimethylamino-<br>methylidene)amino-<br>pentanoate, 2-Amino-<br>5-(amino-<br>dimethylamino-<br>methylidene)amino-<br>pentanoic acid,<br>ADMA, Asymmetric<br>dimethylarginine,<br>Dimethyl-L-arginine,<br>N(Omega),<br>N(omega)-dimethyl-L-<br>arginine, NG, NG-<br>Dimethyl-L-arginine,<br>NG, NG-<br>dimethylarginine, NG-<br>dimethylarginine,<br>Nomega, Nomega'-<br>Dimethyl-L-arginine<br>2-Amino-5-(amino-<br>dimethylamino-<br>methylidene)amino-<br>pentanoate, 2-Amino-<br>5-(amino-<br>dimethylamino-<br>methylidene)amino-<br>pentanoic acid,<br>ADMA, Asymmetric<br>dimethylarginine,<br>Dimethyl-L-arginine,<br>N(Omega),<br>N(omega)-dimethyl-L-	HMDB01539<br>HMDB01539	C03626<br>C03626	Amino Acids and Derivatives<br>Amino Acids and Derivatives

		Carboxyethylamine, 3-Amino-Propanoate, 3-Amino-Propanoic acid, 3-Aminopropanoate, 3-Aminopropanoic acid, 3-Aminopropionate, 3-Aminopropionic acid, Abufene, B-Alanine, b-Aminopropanoate, b-Aminopropanoic acid, b-Aminopropionate, b-Aminopropionic acid, beta Alanine, beta-Alanine, beta-Aminopropanoate, beta-Aminopropanoic acid, beta-Aminopropionate, beta-Aminopropionic acid, Omega-Aminopropionate, Omega-Aminopropionic acid			
	beta-Alanine	(2S)-2-	HMDB00056	C00099	Amino Acids and Derivatives
	L-Alanine	Aminopropanoate,	HMDB00161	C00041	Amino Acids and Derivatives
	D-Alanine	(2S)-2-	HMDB01310	C00133	Amino Acids and Derivatives
	beta-Alanine	Aminopropanoic	HMDB00056	C00099	Amino Acids and Derivatives
	L-Alanine	acid, (S)-(+)-Alanine,	HMDB00161	C00041	Amino Acids and Derivatives
Ala	D-Alanine	(S)-2-amino-	HMDB01310	C00133	Amino Acids and Derivatives

	L-2-Aminoadipic acid	(+/-)-2-Aminoadipic acid, 2-Aminoadipate, 2-Aminoadipic acid, a-Aminoadipate, a-Aminoadipic acid, alpha-Amino-adipic acid, alpha-Aminoadipate, alpha-Aminoadipic acid, Aminoadipate, DL-2-Aminoadipate, DL-2-Aminoadipic acid, DL-2-Aminohexanedioate, DL-2-Aminohexanedioic acid, DL-a-Aminoadipate, DL-a-Aminoadipic acid, DL-alpha-Aminoadipate, DL-alpha-Aminoadipic acid, L-2-Aminoadipate, L-2-Aminoadipic acid, L-2-Aminohexanedioate, L-2-Aminohexanedioic acid, L-alpha-Aminoadipate, L-	HMDB00510	C00956	Amino Acids and Derivatives
alpha-AAA	L-2-Aminoadipic acid	alpha-Aminoadipic	HMDB00510	C00956	Amino Acids and Derivatives

	L-Arginine	[(aminoiminomethyl) amino]-Pentanoate, (S)-2-amino-5-[(aminoiminomethyl) amino]-Pentanoic acid, (S)-2-Amino-5-[(aminoiminomethyl) amino]pentanoate, (S)-2-Amino-5-[(aminoiminomethyl) amino]pentanoic acid, 2-Amino-5-guanidinovalerate, 2-Amino-5-guanidinovaleric acid, 5-[(Aminoiminomethyl) amino]-L-Norvaline, Arginine, L-(+)-Arginine, L-a-Amino-D-guanidinovalerate, L-a-Amino-D-guanidinovaleric acid, L-alpha-Amino-delta-guanidinovalerate, L-alpha-Amino-delta-guanidinovaleric acid, N5-	HMDB00517	C00062	Amino Acids and Derivatives
	D-Arginine	(aminoiminomethyl)-	HMDB03416	C00792	Amino Acids and Derivatives
	L-Arginine	L-Ornithine	HMDB00517	C00062	Amino Acids and Derivatives
Arg	D-Arginine	D-2-Amino-5-	HMDB03416	C00792	Amino Acids and Derivatives

Asn	L-Asparagine<br>D-Asparagine<br>L-Asparagine<br>D-Asparagine	4-Diamino-4-oxobutanoate, (S)-2, 4-Diamino-4-oxobutanoic acid, (S)-Asparagine, 2-Aminosuccinamate, 2-Aminosuccinamic acid, a-Aminosuccinamate, a-Aminosuccinamic acid, Agedoite, alpha Amminosuccinamate, alpha Amminosuccinamic acid, alpha-Aminosuccinamate, alpha-Aminosuccinamic acid, Altheine, Asn, Asparagine, Asparagine acid, Asparamide, Aspartamate, Aspartamic acid, Aspartic acid amide, Aspartic acid b-amide, Aspartic acid beta amide, B2, 4-(S)-diamino-4-oxo-utanoate, B2, 4-(S)-diamino-4-oxo-utanoic acid, Crystal	HMDB00168<br>HMDB33780<br>HMDB00168<br>HMDB33780	C00152<br>C01905<br>C00152<br>C01905	Amino Acids and Derivatives<br>Amino Acids and Derivatives<br>Amino Acids and Derivatives<br>Amino Acids and Derivatives

Asp	L-Aspartic acid<br>D-Aspartic acid<br>L-Aspartic acid<br>D-Aspartic acid	(+)-Aspartic acid, (2S)-Aspartate, (2S)-Aspartic acid, (L)-Aspartate, (L)-Aspartic acid, (R)-2-aminosuccinate, (S)-(+)-Aspartate, (S)-(+)-Aspartic acid, (S)-2-aminosuccinate, (S)-2-aminosuccinic acid, (S)-amino-Butanedioate, (S)-amino-Butanedioic acid, (S)-Aminobutanedioate, (S)-Aminobutanedioic acid, (S)-Aspartate, (S)-Aspartic acid, 2-Amino-3-methylsuccinate, 2-Amino-3-methylsuccinic acid, 2-Aminosuccinate, 2-Aminosuccinic acid, alpha-Aminosuccinate, alpha-Aminosuccinic acid, Aminosuccinate, Asparagate, Asparagic acid,	HMDB00191<br>HMDB06483<br>HMDB00191<br>HMDB06483	C00049<br>C00402<br>C00049<br>C00402	Amino Acids and Derivatives<br>Amino Acids and Derivatives<br>Amino Acids and Derivatives<br>Amino Acids and Derivatives

	L-Carnitine	(trimethylammonio)butyrate, (-)-carnitine, (R)-(3-Carboxy-2-hydroxypropyl)trimethylammonium hydroxide, (R)-carnitine, (S)-carnitine, 1-Carnitine, 3-Carboxy-2-hydroxy-N, N, N-trimethyl-1-propanaminium, 3-Hydroxy-4-trimethylammoniobutanoate, 3-Hydroxy-4-trimethylammoniobutanoic acid, Bicarnesine, Carniking, Carniking 50, Carnilean, Carnipass, Carnipass 20, Carnitene, Carnitine, Carnitor, D-Carnitine, delta-Carnitine, DL-carnitine, gamma-Trimethyl-ammonium-beta-hydroxybutirate, gamma-Trimethyl-beta-hydroxybutyrobetaine, gamma-Trimethyl-	HMDB00062	C15025	Alkylamines
C0	D-Carnitine	hydroxybutyrobetaine	HMDB00062	C15025	Alkylamines

C10	Decanoylcarnitine<br>Decanoyl-L-carnitine<br>Decanoylcarnitine	Decanoyl-L-carnitine,<br>Decanoylcarnitine<br>Decanoyl-L-carnitine,<br>Decanoylcarnitine<br>Decanoyl-L-carnitine,<br>Decanoylcarnitine	HMDB00651<br>HMDB00651<br>HMDB00651		Fatty Acid Esters<br>Fatty Acid Esters<br>Fatty Acid Esters
	C10:1		HMDB13205		Fatty Acid Esters
	C10:2				

C12	Dodecanoylcarnitine<br>Dodecanoyl-L-carnitine<br>Dodecanoylcarnitine	(-)-Lauroylcarnitine, (R)-Dodecanoylcarnitine, Dodecanoyl-L-carnitine, L-Carnitine dodecanoyl ester, Lauroyl-L(-)-carnitin, Lauroyl-L-carnitine, Lauroylcarnitine<br>(-)-Lauroylcarnitine, (R)-Dodecanoylcarnitine, Dodecanoyl-L-carnitine, L-Carnitine dodecanoyl ester, Lauroyl-L(-)-carnitin, Lauroyl-L-carnitine, Lauroylcarnitine<br>(-)-Lauroylcarnitine, (R)-Dodecanoylcarnitine, Dodecanoyl-L-carnitine, L-Carnitine dodecanoyl ester, Lauroyl-L(-)-carnitin, Lauroyl-L-carnitine, Lauroylcarnitine	HMDB02250<br>HMDB02250<br>HMDB02250		Fatty Acid Esters<br>Fatty Acid Esters<br>Fatty Acid Esters
C12-DC	Dodecanedioylcarnitine<br>Dodecanedioylcarnitine	Dodecanedioyl-L-carnitine<br>Dodecanedioyl-L-carnitine	HMDB13327<br>HMDB13327		Carboxylic Acids and Derivatives<br>Carboxylic Acids and Derivatives
C12:1	C12:1				

C14	C14	(-)-Myristoylcarnitine,	HMDB05066		Fatty Acid Esters
C14:1	cis-5-Tetradecenoylcarnitine cis-5-Tetradecenoylcarnitine		HMDB02014		Fatty Acid Esters
C14:1-OH	C14:1-OH	3-Hydroxy-5(Z)-tetrad	HMDB13330		Hydroxy Acids and Derivatives
C14:2	C14:2	(3E, 5E)-tetradecadie	HMDB13331		Fatty Acid Esters
C14:2-OH	C14:2-OH				

C16	L-Palmitoylcarnitine<br>D-Palmitoylcarnitine<br>L-Palmitoylcarnitine<br>Palmitoylcarnitine	palmitoylcarnitine, (3S)-3-hexadecanoyloxy-4-(trimethylammonio)butanoate, (3S)-3-hexadecanoyloxy-4-(trimethylammonio)butanoic acid, (3S)-3-palmitoyloxy-4-(trimethylammonio)butanoate, (3S)-3-palmitoyloxy-4-(trimethylammonio)butanoic acid, 3-Carboxy-N, N, N-trimethyl-2-[(1-oxohexadecyl)oxy]-1-Propanaminium, D-Palmitylcarnitine, Hexadecanoyl-L-carnitine, Hexadecenoyl carnitine, L(-)-Palmitylcarnitine, L-Carnitine palmitoyl ester, L-Palmitoyl-L-carnitine, Palmitoyl D-carnitine, Palmitoyl-(-)-carnitine, Palmitoyl-L-carnitine, Palmityl-L-carnitine<br>(+)-	HMDB00222<br>HMDB00222<br>HMDB00222<br>HMDB00222	C02990<br>C02990<br>C02990<br>C02990	Fatty Acid Esters<br>Fatty Acid Esters<br>Fatty Acid Esters<br>Fatty Acid Esters
C16-OH	C16-OH				

C16:1	trans-Hexadec-2-enoyl carnitine trans-Hexadec-2-enoyl carnitine	(2E)-Hexadecenoyl-carnitine, trans-2-Hexadecenoyl-carnitine (2E)-Hexadecenoyl-carnitine, trans-2-Hexadecenoyl-carnitine	HMDB06317 HMDB06317		Fatty Acid Esters Fatty Acid Esters
C16:1-OH	C16:1-OH	3-Hydroxy-(9Z)-hexad	HMDB13333		Hydroxy Acids and Derivatives
C16:2	C16:2	(Z, Z)9, 12-hexadecad	HMDB13334		Fatty Acid Esters
C16:2-OH	C16:2-OH	3-Hydroxy-9(Z), 12(Z)	HMDB13335		Hydroxy Acids and Derivatives

C18	Stearoylcarnitine<br>Stearoylcarnitine	(R)-3-carboxy-N, N, N-trimethyl-2-[(1-oxooctadecyl)oxy]-1-Propanaminium inner salt, (R)-Stearoylcarnitine, Acylcarnitine C18:0, L-(3-Carboxy-2-hydroxypropyl)trimethyl-Ammonium stearate hydroxide inner salt, L-Stearic acid ester with (3-carboxy-2-hydroxypropyl)trimethylammonium hydroxide inner salt, L-Stearoylcarnitine, Octadecanoyl-L-carnitine, Octadecanoylcarnitine, Stearoyl-L-carnitine, Stearoylcarnitine (-)-Stearoylcarnitine, (R)-3-carboxy-N, N, N-trimethyl-2-[(1-oxooctadecyl)oxy]-1-Propanaminium inner salt, (R)-Stearoylcarnitine, Acylcarnitine C18:0,	HMDB00848<br>HMDB00848		Fatty Acid Esters<br>Fatty Acid Esters

C18:1	Oleoylcarnitine<br>Elaidic carnitine<br>Elaidic carnitine<br>Oleoylcarnitine	(Z)-(+-)-3-carboxy-N, N, N-trimethyl-2-((1-oxo-9-octadecenyl)oxy)-1-Propanaminium, Acylcarnitine C18:1, Oleoyl-L-carnitine, Oleoylcarnitine	HMDB05065<br>HMDB06464<br>HMDB06464<br>HMDB06464		Fatty Acid Esters<br>Fatty Acid Esters<br>Fatty Acid Esters<br>Fatty Acid Esters
C18:1-OH	C18:1-OH	3-Hydroxy-11(Z)-octad	HMDB13339		Hydroxy Acids and Derivatives
C18:2	Linoelaidylcarnitine	Acylcarnitine C18:2	HMDB06461		Fatty Acid Esters

	Acetyl-L-carnitine	(+-)-Acetylcarnitine, (-)-Acetylcarnitine, (R)-Acetylcarnitine, Acetyl-carnitine , Acetyl-L-(-)-carnitine, Acetyl-L-carnitine, Acetylcarnitine, ALCAR, L-Acetylcarnitine, L-Carnitine acetyl ester, L-O-Acetylcarnitine, Levocarnitine acetyl, Nicetile, O-Acetyl-L-carnitine, O-Acetylcarnitine	HMDB00201	C02571	Fatty Acid Esters
C2	Acetylcarnitine	(+-)-Acetylcarnitine, (-)-Acetylcarnitine, (R)-Acetylcarnitine, Acetyl-carnitine , Acetyl-L-(-)-carnitine, Acetyl-L-carnitine, Acetylcarnitine, ALCAR, L-Acetylcarnitine, L-Carnitine acetyl ester, L-O-Acetylcarnitine, Levocarnitine acetyl, Nicetile, O-Acetyl-L-carnitine, O-Acetylcarnitine	HMDB00201	C02571	Fatty Acid Esters

		Propionylcarnitine chloride, (3-carboxy-2-hydroxypropyl)trimethyl-hydroxide Ammonium inner salt, 3-Carboxy-N, N, N-trimethyl-2-(1-oxopropoxy)-1-propanaminium inner salt, L-Propionylcarnitine, O-Propanoylcarnitine, O-Propionylcarnitine, Propionyl carnitine, Propionyl-carnitine, Propionyl-L-carnitine (+/-)-Propionylcarnitine chloride, (3-carboxy-2-hydroxypropyl)trimethyl-hydroxide Ammonium inner salt, 3-Carboxy-N, N, N-trimethyl-2-(1-oxopropoxy)-1-propanaminium inner salt, L-			
	Propionylcarnitine	Propionylcarnitine, O-	HMDB00824	C03017	Fatty Acid Esters
	Propionyl-L-carnitine	Propanoylcarnitine,	HMDB00824	C03017	Fatty Acid Esters
C3	Propionylcarnitine	O-Propionylcarnitine,	HMDB00824	C03017	Fatty Acid Esters

		Carboxyacetyl)oxy]-4-hydroxy-N, N, N-trimethyl-4-oxo-1-Butanaminium inner salt, 3-Carboxy-2-[(carboxyacetyl)oxy]-N, N, N-trimethyl-1-Propanaminium inner salt, Malonyl-L-carnitine<br><br>2-[(2-Carboxyacetyl)oxy]-4-hydroxy-N, N, N-trimethyl-4-oxo-1-Butanaminium inner salt, 3-Carboxy-2-[(carboxyacetyl)oxy]-N, N, N-trimethyl-1-Propanaminium inner salt, Malonyl-L-carnitine<br>2-[(2-Carboxyacetyl)oxy]-4-hydroxy-N, N, N-trimethyl-4-oxo-1-Butanaminium inner salt, 3-Carboxy-2-[(carboxyacetyl)oxy]-N, N, N-trimethyl-1-Propanaminium			
C3-DC (C4-OH)	Malonylcarnitine<br>Hydroxybutyrylcarnitine<br>Malonyl-D-carnitine<br>Malonyl-L-carnitine<br>Malonylcarnitine<br>Hydroxybutyrylcarnitine		HMDB02095<br>HMDB13127<br>HMDB02095<br>HMDB02095<br>HMDB02095<br>HMDB13127		Fatty Acid Esters<br>Fatty Acid Esters<br>Fatty Acid Esters<br>Fatty Acid Esters<br>Fatty Acid Esters<br>Fatty Acid Esters
C3-OH	Hydroxypropionylcarn	Hydroxypropionyl-L-ca	HMDB13125		Fatty Acid Esters
C3:1	C3:1	Propenoyl-L-carnitine	HMDB13124		Fatty Acid Esters

		carnitine, Isobutyryl-L-carnitine, L-Isobutyric acid ester with (3-carboxy-2-hydroxypropyl)trimethylammonium hydroxide inner salt (3-carboxy-2-hydroxypropyl)trimethyl-L-butyrate, (R)-3-carboxy-N, N, N-trimethyl-2-(1-oxobutoxy)-1-Propanaminium, Butyryl-L-carnitine, Ester with (3-carboxy-2-hydroxypropyl)trimethylammonium L-Butyric acid, Ester with L-(3-carboxy-2-hydroxypropyl)trimethylammonium Butyric acid, L-Carnitine butyryl ester, N-Butyryl-L(-)-			
	Isobutyryl-L-carnitine	carnitine, O-	HMDB00736		Fatty Acid Esters
	Butyrylcarnitine	Butanoylcarnitine	HMDB02013	C02862	Fatty Acid Esters
	Isobutyryl-L-carnitine	Isobutyryl-L-(-)-	HMDB00736		Fatty Acid Esters
	Isobutyrylcarnitine	carnitine, Isobutyryl-L-	HMDB00736		Fatty Acid Esters
	Butyryl-L-carnitine	carnitine, L-Isobutyric	HMDB02013	C02862	Fatty Acid Esters
C4	Butyrylcarnitine	acid ester with (3-	HMDB02013	C02862	Fatty Acid Esters
C4:1	Butenyl-L-carnitine	Butenyl-L-carnitine	HMDB13126		Fatty Acid Esters

Supplement table 1

C5	Isovalerylcarnitine<br>Valerylcarnitine<br>Pivaloylcarnitine<br>2-Methylbutyrylcarnitine<br>Isovalery-L-carnitine<br>Isovalerylcarnitine<br>Valeryl-L-carnitine<br>Valerylcarnitine	Isovaleryl L-carnitine, Isovalerylcarnitine Pentanoylcarnitine, Valeryl-L-carnitine<br><br>(2-methylbutyryl)carnitine, 2-Methylbutyroylcarnitine, L-2-Methylbutyrate (3-carboxy-2-hydroxypropyl)trimethyl-hydroxide Ammonium inner salt, Methylbutyroyl-carnitine<br>Isovaleryl L-carnitine, Isovalerylcarnitine<br>Isovaleryl L-carnitine, Isovalerylcarnitine<br>Pentanoylcarnitine, Valeryl-L-carnitine<br>Pentanoylcarnitine, Valeryl-L-carnitine	HMDB00688<br>HMDB13128<br>HMDB41993<br>HMDB00378<br>HMDB00688<br>HMDB00688<br>HMDB13128<br>HMDB13128		Fatty Acid Esters<br>Fatty Acid Esters<br>Fatty Acid Esters<br>Fatty Acid Esters<br>Fatty Acid Esters<br>Fatty Acid Esters<br>Fatty Acid Esters<br>Fatty Acid Esters
C5-DC (C6-OH)	C5-DC (C6-OH)		HMDB13130		Fatty Acid Esters
C5-M-DC	Methylglutaryl-L-carnit	3-Methylglutarylcarniti	HMDB00552		Fatty Acid Esters
C5-OH (C3-DC-M)	C5-OH (C3-DC-M)		HMDB13132		Alkylamines
C5:1	Tiglylcarnitine<br>Tiglylcarnitine	Tiglyl-L-carnitine<br>Tiglyl-L-carnitine	HMDB02366<br>HMDB02366		Fatty Acid Esters<br>Fatty Acid Esters
C5:1-DC	Glutaconylcarnitine	Glutaconyl-L-carnitine	HMDB13129		Fatty Acid Esters

C6 (C4:1-DC)	Hexanoylcarnitine (Fumarylcarnitine) L-Hexanoylcarnitine Hexanoylcarnitine (Fumarylcarnitine) L-Hexanoylcarnitine	Hexanoate, Hexanoic acid, Hexanoic acid ester with (3-carboxy-2-hydroxypropyl)trimethylammonium hydroxide inner salt, Hexanoyl DL-carnitine, Hexanoyl-D, L-carnitine, Hexanoylcarnitine (R)-3-carboxy-N, N, N-trimethyl-2-[(1-oxohexyl)oxy]-1-Propanaminium inner salt, (R)-Caproylcarnitine, Hexanoate, Hexanoic acid, Hexanoic acid ester with L-(3-carboxy-2-hydroxypropyl)trimethylammonium hydroxide inner salt, Hexanoyl-L-carnitine, L-Carnitine hexanoyl ester, L-Hexanoate, L-Hexanoic acid, L-Hexanoic acid ester with (3-carboxy-2-hydroxypropyl)trimethylammonium	HMDB00705 HMDB00756 HMDB00705 HMDB00756		Fatty Acid Esters Fatty Acid Esters Fatty Acid Esters Fatty Acid Esters
C6:1	C6:1	Hexenoyl-L-carnitine	HMDB13161		Fatty Acid Esters
C7-DC	Pimelylcarnitine	Heptanedioylcarnitine	HMDB13328		Carboxylic Acids and Derivatives

C8	Octanoyl-L-carnitine<br>Octanoylcarnitine	(-)-Octanoylcarnitine, L-Carnitine octanoyl ester, L-O-Octanoylcarnitine, L-Octanoylcarnitine, Octanoate, Octanoic acid, Octanoic acid ester with L-(3-carboxy-2-hydroxypropyl)trimethylammonium hydroxide inner salt, Octanoyl-L-carnitine, Octanoylcarnitine<br>(-)-Octanoylcarnitine, L-Carnitine octanoyl ester, L-O-Octanoylcarnitine, L-Octanoylcarnitine, Octanoate, Octanoic acid, Octanoic acid ester with L-(3-carboxy-2-hydroxypropyl)trimethylammonium hydroxide inner salt, Octanoyl-L-carnitine, Octanoylcarnitine	HMDB00791<br>HMDB00791	C02838<br>C02838	Fatty Acid Esters<br>Fatty Acid Esters
C9	2,6 Dimethylheptanoy	2, 6-Dimethylheptanoy	HMDB06320		Fatty Acid Esters

		b-Alanylhistidine, beta-Alanyl-L-histidine, beta-Alanylhistidine, Carnosine, Ignotine, Karnozin, Karnozzn, L-Carnosine, N-(3-Aminopropanoyl)histidine, N-(b-Alanyl)-L-histidine, N-b-Alanyl-L-Histidine, N-beta-Alanyl-L-Histidine, Sevitin b-Alanyl-L-histidine, b-Alanylhistidine, beta-Alanyl-L-histidine, beta-Alanylhistidine, Carnosine, Ignotine, Karnozin, Karnozzn, L-Carnosine, N-(3-Aminopropanoyl)histidine, N-(b-Alanyl)-L-histidine, N-b-Alanyl-L-Histidine, N-beta-Alanyl-L-Histidine, Sevitin			
	Carnosine	b-Alanyl-L-histidine,	HMDB00033	C00386	Peptidomimetics
	L-Carnosine	b-Alanylhistidine,	HMDB00033	C00386	Peptidomimetics
	Carnosine	beta-Alanyl-L-	HMDB00033	C00386	Peptidomimetics
	L-Carnosine	histidine, beta-	HMDB00033	C00386	Peptidomimetics
Carnosine	L-Carnosine	Alanylhistidine,	HMDB00033	C00386	Peptidomimetics

Supplement table 1

	Citrulline		HMDB00904	C00327	Amino Acids and Derivatives
	L-Citrulline		HMDB00904	C00327	Amino Acids and Derivatives
Cit	L-Citrulline	(2S)-2-amino-5-(carba	HMDB00904	C00327	Amino Acids and Derivatives

	Creatinine	1-Methylglycocyamidine, 1-Methylhydantoin-2-imide, 2-Amino-1-methyl-1, 5-dihydroimidazol-4-one, 2-Amino-1-methylimidazolin-4-one, Creatine anhydride, Creatinine	HMDB00562	C00791	Lactams
	Creatinine	1-Methylglycocyamidine, 1-Methylhydantoin-2-imide, 2-Amino-1-methyl-1, 5-dihydroimidazol-4-one, 2-Amino-1-methylimidazolin-4-one, Creatine anhydride, Creatinine	HMDB00562	C00791	Lactams
Creatinine	Creatinine	1-Methylglycocyamidine, 1-Methylhydantoin-2-imide, 2-Amino-1-methyl-1, 5-dihydroimidazol-4-one, 2-Amino-1-methylimidazolin-4-one, Creatine anhydride, Creatinine	HMDB00562	C00791	Lactams

Supplement table 1

DOPA	L-Dihydroxyphenylalanine<br>Dihydroxyphenylalanine	(-)-3-(3, 4-dihydroxyph	HMDB00181<br>HMDB00609	C00355<br>C00355	Amino Acids and Derivatives<br>Amino Acids and Derivatives

	Dopamine	Dihydroxyphenyl)ethylamine, 3, 4-Dihydroxyphenethylamine, 3, 4-Dihydroxyphenylethylamine, 3-Hydroxytyramine, 4-(2-Aminoethyl)-1, 2-benzenediol, 4-(2-Aminoethyl)-Pyrocatechol, 4-(2-Aminoethyl)catechol, 4-(2-Aminoethyl)pyrocatechol, a-(3, 4-Dihydroxyphenyl)-b-aminoethane, alpha-(3, 4-Dihydroxyphenyl)-beta-aminoethane, Deoxyepinephrine, Dopamin, Dopamine, Dopaminum, Dopastat, Dophamine, Dynatra, Hydroxytyramin, Hydroxytyramine, Intropin, Oxytyramine, Revivan	HMDB00073	C03758	Phenols and Derivatives
	Dopamine	2-(3, 4-	HMDB00073	C03758	Phenols and Derivatives
Dopamine	Dopamine	Dihydroxyphenyl)ethy	HMDB00073	C03758	Phenols and Derivatives

Gln	L-Glutamine<br>D-Glutamine<br>L-Glutamine<br>D-Glutamine	oxopentanoate, (2S)-2, 5-diamino-5-oxopentanoic acid, (2S)-2-amino-4-carbamoylbutanoate, (2S)-2-amino-4-carbamoylbutanoic acid, (S)-2, 5-Diamino-5-oxopentanoate, (S)-2, 5-Diamino-5-oxopentanoic acid, 2-Aminoglutaramic acid, Cebrogen, gamma-Glutamine, Glavamin, Glumin, Glutamic acid 5-amide, Glutamic acid amide, Glutamine, L-(+)-Glutamine, L-2-Aminoglutaramic acid, L-2-Aminoglutaramidic acid, L-Glutamic acid 5-amide, L-Glutamic acid gamma-amide, L-Glutamid, L-Glutamide, L-Glutamin, L-Glutamine, L-Glutaminsaeure-5-amid, Levoglutamid,	HMDB00641<br>HMDB03423<br>HMDB00641<br>HMDB03423	C00064<br>C00819<br>C00064<br>C00819	Amino Acids and Derivatives<br>Amino Acids and Derivatives<br>Amino Acids and Derivatives<br>Amino Acids and Derivatives

Glu	L-Glutamic acid<br>D-Glutamic acid<br>L-Glutamic acid<br>D-Glutamic acid	Aminopentanedioate, (2S)-2-Aminopentanedioic acid, (S)-(+)-Glutamate, (S)-(+)-Glutamic acid, (S)-2-Aminopentanedioate, (S)-2-Aminopentanedioic acid, (S)-Glutamate, (S)-Glutamic acid, 1-Amino-propane-1, 3-dicarboxylate, 1-Amino-propane-1, 3-dicarboxylic acid, 1-Aminopropane-1, 3-dicarboxylate, 1-Aminopropane-1, 3-dicarboxylic acid, 2-Aminoglutarate, 2-Aminoglutaric acid, 2-Aminopentanedioate, 2-Aminopentanedioic acid, a-Aminoglutarate, a-Aminoglutaric acid, a-Glutamate, a-Glutamic acid, Aciglut, alpha-Aminoglutarate, alpha-Aminoglutaric acid, alpha-	HMDB00148<br>HMDB03339<br>HMDB00148<br>HMDB03339	C00025<br>C00217<br>C00025<br>C00217	Amino Acids and Derivatives<br>Amino Acids and Derivatives<br>Amino Acids and Derivatives<br>Amino Acids and Derivatives

Gly	Glycine<br>Glycine	2-Aminoacetate, 2-Aminoacetic acid, Aciport, Amino-Acetate, Amino-Acetic acid, Aminoacetate, Aminoacetic acid, Aminoethanoate, Aminoethanoic acid, Glicoamin, Glycocoll, Glycolixir, Glycosthene, Gyn-Hydralin, Padil<br>2-Aminoacetate, 2-Aminoacetic acid, Aciport, Amino-Acetate, Amino-Acetic acid, Aminoacetate, Aminoacetic acid, Aminoethanoate, Aminoethanoic acid, Glicoamin, Glycocoll, Glycolixir, Glycosthene, Gyn-Hydralin, Padil	HMDB00123<br>HMDB00123	C00037<br>C00037	Amino Acids and Derivatives<br>Amino Acids and Derivatives

		Glucose, Anhydrous			
		dextrose, Cerelose,			
		Cerelose 2001,			
		Clearsweet 95,			
		Clintose L, Corn			
		sugar, CPC hydrate,			
		D(+)-Glucose,			
		Dextropur, Dextrose,			
		Dextrosol, Glucodin,			
	D-Glucose	Glucolin, Glucose,	HMDB00122	C00031	Monosaccharides
	D-Galactose	Goldsugar, Grape	HMDB00143	C00984	Monosaccharides
	D-Galactose	sugar, Meritose,	HMDB00143	C00984	Monosaccharides
	D-Mannose	Staleydex 111,	HMDB00169	C00159	Monosaccharides
	Beta-D-Glucose	Staleydex 95M,	HMDB00516	C00221	Monosaccharides
	D-Fructose	Tabfine 097(HS),	HMDB00660	C02336	Monosaccharides
	D-Fructose	Vadex	HMDB00660	C02336	Monosaccharides
	L-Sorbose	(+)-Galactose, 5abp,	HMDB01266	C08356	Monosaccharides
	Alpha-D-Glucose	8abp, alpha D-	HMDB03345	C00267	Monosaccharides
	D-Tagatose	Galactose, alpha-D-	HMDB03418	C00795	Monosaccharides
	Beta-D-Galactose	Galactopyranose,	HMDB03449	C00962	Monosaccharides
	L-Gulose	alpha-D-Galactose,	HMDB12326	C15923	Monosaccharides
	L-Gulose	D-(+)-Galactose, D-	HMDB12326	C15923	Monosaccharides
	L-Galactose	Galactose, D-	HMDB33704	C01825	Monosaccharides
	D-Glucose	Hexose, GAL,	HMDB00122	C00031	Monosaccharides
	D-Galactose	Galactose,	HMDB00143	C00984	Monosaccharides
	D-Mannose	Galactose (NF),	HMDB00169	C00159	Monosaccharides
	Beta-D-Glucose	GLA, GLC, Hexose	HMDB00516	C00221	Monosaccharides
	D-Fructose	(+)-Galactose, 5abp,	HMDB00660	C02336	Monosaccharides
	Alpha-D-Glucose	8abp, alpha D-	HMDB03345	C00267	Monosaccharides
	D-Tagatose	Galactose, alpha-D-	HMDB03418	C00795	Monosaccharides
	Beta-D-Galactose	Galactopyranose,	HMDB03449	C00962	Monosaccharides
H1	L-Gulose	alpha-D-Galactose,	HMDB12326	C15923	Monosaccharides

	L-Histidine	alanine, (S)-2-Amino-3-(4-imidazolyl)propionsaeure, (S)-4-(2-Amino-2-carboxyethyl)imidazole, (S)-a-Amino-1H-imidazole-4-propanoate, (S)-a-Amino-1H-imidazole-4-propanoic acid, (S)-alpha-Amino-1H-imidazole-4-propanoate, (S)-alpha-Amino-1H-imidazole-4-propanoic acid, (S)-alpha-Amino-1H-imidazole-4-propionate, (S)-alpha-Amino-1H-imidazole-4-propionic acid, (S)-Histidine, (S)1H-Imidazole-4-alanine, 3-(1H-Imidazol-4-yl)-L-Alanine, Amino-1H-imidazole-4-propanoate, Amino-1H-imidazole-4-propanoic acid, Amino-4-	HMDB00177	C00135	Amino Acids and Derivatives
His	L-Histidine	imidazoleproprionate,	HMDB00177	C00135	Amino Acids and Derivatives

		ethanamine, 2-(1H-Imidazol-4-yl)ethanamine, 2-(1H-Imidazol-4-yl)ethylamine, 2-(1H-Imidazol-5-yl)ethanamine, 2-(1H-Imidazol-5-yl)ethylamine, 2-(4-Imidazolyl)ethanamine, 2-(4-Imidazolyl)ethylamine, 2-Imidazol-4-yl-Ethylamine, 2-Imidazol-4-ylethylamine, 4-(2-Aminoethyl)-1H-imidazole, 4-(2-Aminoethyl)imidazole, 4-Imidazoleethylamine, 5-Imidazoleethylamine, b-Imidazolyl-4-ethylamine, beta-Aminoethylglyoxaline, beta-Aminoethylimidazole, beta-			
	Histamine	Aminothethylglyoxali	HMDB00870	C00388	Azoles
	Histamine	ne, beta-Imidazolyl-4-	HMDB00870	C00388	Azoles
Histamine	Histamine	ethylamine, Eramin,	HMDB00870	C00388	Azoles

Supplement table 1

	L-Isoleucine		HMDB00172		Amino Acids and Derivatives
	L-Isoleucine		HMDB00172	C00407	Amino Acids and Derivatives
Ile	Allo-L-Isoleucine	(2S, 3S)-2-amino-3-m	HMDB00557	C00407	Amino Acids and Derivatives

		diamino-3-hydroxy-gamma-oxo-Benzenebutanoate, (alphaS)-alpha, 2-diamino-3-hydroxy-gamma-oxo-Benzenebutanoic acid, (S)-alpha, 2-diamino-3-hydroxy-gamma-oxo-Benzenebutanoate, (S)-alpha, 2-diamino-3-hydroxy-gamma-oxo-Benzenebutanoic acid, 3-(3-Hydroxyanthraniloyl)-L-alanine, 3-Anthraniloyl-Alanine, 3-Anthraniloyl-L-alanine, 3-Anthraniloylalanine, 3-Hydroxy-L-kynurenine, alpha, 2-Diamino-gamma-oxo-Benzenebutanoate, alpha, 2-Diamino-gamma-oxo-Benzenebutanoic acid, Dl-Kynurenine,			
	L-Kynurenine	DL-Kynureninefree	HMDB00684	C00328	Amino Acids and Derivatives
Kynurenine	L-Kynurenine	base, Kynurenin,	HMDB00684	C00328	Amino Acids and Derivatives

		methylpentanoate, (2S)-2-Amino-4-methylpentanoic acid, (S)-(+)-Leucine, (S)-2-Amino-4-methylpentanoate, (S)-2-Amino-4-methylpentanoic acid, (S)-2-Amino-4-methylvalerate, (S)-2-Amino-4-methylvaleric acid, (S)-Leucine, 4-Methyl-L-Norvaline, L-(+)-Leucine, L-a-Aminoisocaproate, L-a-Aminoisocaproic acid, L-alpha-Aminoisocaproate, L-alpha-Aminoisocaproic acid, Leu, Leucine			
	L-Leucine	beta-2-Amino-4-methylvaleric acid, beta-Leucine, L-beta-Leucine	HMDB00687	C00123	Amino Acids and Derivatives
	beta-Leucine		HMDB03640	C02486	Amino Acids and Derivatives
	D-beta-Leucine	beta-2-Amino-4-	HMDB03640	C02486	Amino Acids and Derivatives
	L-beta-Leucine	methylvaleric acid,	HMDB03640	C02486	Amino Acids and Derivatives
	D-Leucine	beta-Leucine, L-beta-	HMDB13773	C01570	Amino Acids and Derivatives
	L-Leucine	Leucine	HMDB00687	C00123	Amino Acids and Derivatives
	D-beta-Leucine	beta-2-Amino-4-	HMDB03640	C02486	Amino Acids and Derivatives
Leu	L-beta-Leucine	methylvaleric acid,	HMDB03640	C02486	Amino Acids and Derivatives

Lys	L-Lysine<br>D-Lysine<br>L-Lysine<br>D-Lysine	diamino-Hexanoate, (S)-2, 6-diamino-Hexanoic acid, (S)-2, 6-Diaminohexanoate, (S)-2, 6-Diaminohexanoic acid, (S)-a, e-Diaminocaproate, (S)-a, e-Diaminocaproic acid, (S)-Lysine, 2, 6-Diaminohexanoate, 2, 6-Diaminohexanoic acid, 6-Amino-Aminutrin, 6-Amino-L-Norleucine, a-Lysine, alpha-Lysine, Aminutrin, h-Lys-oh, L-(+)-Lysine, L-2, 6-Diainohexanoate, L-2, 6-Diainohexanoic acid, L-2, 6-Diaminocaproate, L-2, 6-Diaminocaproic acid, L-Lys, Lys, Lysine, Lysine acid<br>D-2, 6-Diaminohexanoate, D-2, 6-Diaminohexanoic acid<br>(+)-S-Lysine, (S)-2, 6-	HMDB00182<br>HMDB03405<br>HMDB00182<br>HMDB03405	C00047<br>C00739<br>C00047<br>C00739	Amino Acids and Derivatives<br>Amino Acids and Derivatives<br>Amino Acids and Derivatives<br>Amino Acids and Derivatives

	L-Methionine		HMDB00696	C00073	Amino Acids and Derivatives
Met	L-Methionine	(2S)-2-amino-4-(meth	HMDB00696	C00073	Amino Acids and Derivatives

Met-SO	L-Methionine sulfoxide<br>L-Methionine sulfoxide	2-Amino-4-(methylsulfinyl)-Butanoate, 2-Amino-4-(methylsulfinyl)-Butanoic acid, alpha-Amino-gamma-(methylsulfinyl)-Butyric acid, DL-Methionine sulfoxide, L-Methionine (S)-S-oxide, L-Methionine R-oxide, L-Methionine sulfoxide, Met-SO, S-Oxide-methionine<br>2-Amino-4-(methylsulfinyl)-Butanoate, 2-Amino-4-(methylsulfinyl)-Butanoic acid, alpha-Amino-gamma-(methylsulfinyl)-Butyric acid, DL-Methionine sulfoxide, L-Methionine (S)-S-oxide, L-Methionine R-oxide, L-Methionine sulfoxide, Met-SO, S-Oxide-methionine	HMDB02005<br>HMDB02005		Amino Acids and Derivatives<br>Amino Acids and Derivatives

Nitro-Tyr	3-Nitrotyrosine<br>3-Nitrotyrosine	3-Nitrotyrosine, 5-Nitrotyrosine, m-Nitrotyrosine<br>3-Nitrotyrosine, 5-Nitrotyrosine, m-Nitrotyrosine	HMDB01904<br>HMDB01904		Amino Acids and Derivatives<br>Amino Acids and Derivatives

OH-Pro	4-Hydroxyproline<br>3-Hydroxy-L-proline<br>4-Hydroxy-L-proline<br>cis-4-Hydroxy-L-proline<br>4-Hydroxy-L-proline<br>4-Hydroxyproline<br>3-Hydroxy-L-proline<br>cis-4-Hydroxy-L-proline	pyrrolidinecarboxylic acid, (2S, 4R)-4-hydroxypyrrolidine-2-carboxylic acid, (4R)-4-hydroxy-L-proline, 4-Hydroxy-2-pyrrolidinecarboxylic acid, 4-Hydroxy-L-proline, 4-L-Hydroxyproline, delta-Hydroxyproline, Hydroxiproline, Hydroxy-L-proline, Hydroxy-proline, Hydroxyproline, L-4-Hydroxyproline, L-Hydroxyproline, L-Threo-4-hydroxyproline, LS-hydroxyproline, Oxaceprol, trans-4-Hydroxy-L-proline, trans-4-Hydroxyproline, Trans-hydroxyproline, Trans-L-hydroxyproline<br>Procollagen trans-3-hydroxy-L-proline<br>(2S)-4-hydroxypyrrolidine-2-	HMDB00725<br>HMDB02113<br>HMDB06055<br>HMDB06055<br>HMDB00725<br>HMDB00725<br>HMDB02113<br>HMDB06055	C01157<br>C04397<br>C01015<br>C01015<br>C01157<br>C01157<br>C04397<br>C01015	Amino Acids and Derivatives<br>Amino Acids and Derivatives<br>Amino Acids and Derivatives<br>Amino Acids and Derivatives<br>Amino Acids and Derivatives<br>Amino Acids and Derivatives<br>Amino Acids and Derivatives<br>Amino Acids and Derivatives

Orn	Ornithine<br>L-Ornithine<br>D-Ornithine<br>L-Ornithine<br>D-Ornithine	2, 5-Diaminopentanoate, (S)-2, 5-Diaminopentanoic acid, (S)-a, D-Diaminovalerate, (S)-a, D-Diaminovaleric acid, (S)-Ornithine, 5-Amino-L-Norvaline, L-(-)-Ornithine, L-Ornithine<br>(+)-S-Ornithine, (S)-2, 5-Diaminopentanoate, (S)-2, 5-Diaminopentanoic acid, (S)-a, D-Diaminovalerate, (S)-a, D-Diaminovaleric acid, (S)-Ornithine, 5-Amino-L-Norvaline, L-(-)-Ornithine, L-Ornithine<br>(2R)-2, 5-diaminopentanoate, (2R)-2, 5-diaminopentanoic acid, (R)-ornithine, Ornithine<br>(+)-S-Ornithine, (S)-2, 5-Diaminopentanoate,	HMDB00214<br>HMDB00214<br>HMDB03374<br>HMDB00214<br>HMDB03374	C00077<br>C00077<br>C00515<br>C00077<br>C00515	Amino Acids and Derivatives<br>Amino Acids and Derivatives<br>Amino Acids and Derivatives<br>Amino Acids and Derivatives<br>Amino Acids and Derivatives
PC aa C26:0	PC aa C26:0				
PC aa C28:1	PC aa C28:1				

PC aa C30:0	PC(14:0/16:0)<br>PC(15:0/15:0)<br>PC(16:0/14:0)<br>PC(14:0/16:0)<br>PC(15:0/15:0)<br>PC(16:0/14:0)	palmitoyl-sn-glycero-3-phosphocholine, GPCho(14:0/16:0), GPCho(30:0), Lecithin, PC aa C30:0, PC(14:0/16:0), PC(30:0), Phosphatidylcholine(14:0/16:0), Phosphatidylcholine(30:0)<br>1, 2-Dipentadecanoyl-rac-glycero-3-phosphocholine, GPCho(15:0/15:0), GPCho(30:0), Lecithin, PC aa C30:0, PC(15:0/15:0), PC(30:0), Phosphatidylcholine(15:0/15:0), Phosphatidylcholine(30:0)<br>1-Palmitoyl-2-myristoyl-sn-glycero-3-phosphocholine, GPCho(16:0/14:0), GPCho(30:0), Lecithin, PC aa C30:0,	HMDB07869<br>HMDB07934<br>HMDB07965<br>HMDB07869<br>HMDB07934<br>HMDB07965	C00157<br>C00157<br>C00157<br>C00157<br>C00157<br>C00157	Glycerophospholipids<br>Glycerophospholipids<br>Glycerophospholipids<br>Glycerophospholipids<br>Glycerophospholipids<br>Glycerophospholipids
PC aa C30:2	PC aa C30:2				

Supplement table 1

	PC(16:0/16:0)		HMDB00564	C00157	Glycerophospholipids
	PC(14:0/18:0)		HMDB07871	C00157	Glycerophospholipids
	PC(18:0/14:0)		HMDB08031	C00157	Glycerophospholipids
	PC(16:0/16:0)		HMDB00564	C00157	Glycerophospholipids
	PC(14:0/18:0)		HMDB07871	C00157	Glycerophospholipids
PC aa C32:0	PC(18:0/14:0)	(R)-4-hydroxy-N, N, N	HMDB08031	C00157	Glycerophospholipids

PC aa C32:1	PC(14:0/18:1(11Z))<br>PC(14:0/18:1(9Z))<br>PC(16:0/16:1(9Z))<br>PC(18:1(9Z)/14:0)<br>PC(14:0/18:1(11Z))<br>PC(14:0/18:1(9Z))<br>PC(16:0/16:1(9Z))<br>PC(18:1(9Z)/14:0)	vaccenoyl-sn-glycero-3-phosphocholine, GPCho(14:0/18:1), GPCho(14:0/18:1n7), GPCho(14:0/18:1w7), GPCho(32:1), Lecithin, PC aa C32:1, PC(14:0/18:1), PC(14:0/18:1n7), PC(14:0/18:1w7), PC(32:1), Phosphatidylcholine(14:0/18:1), Phosphatidylcholine(14:0/18:1n7), Phosphatidylcholine(14:0/18:1w7), Phosphatidylcholine(32:1)<br>1-Myristoyl-2-oleoyl-sn-glycero-3-phosphocholine, GPCho(14:0/18:1), GPCho(14:0/18:1n9), GPCho(14:0/18:1w9), GPCho(32:1), Lecithin, PC aa C32:1, PC(14:0/18:1), PC(14:0/18:1n9), PC(14:0/18:1w9),	HMDB07872<br>HMDB07873<br>HMDB07969<br>HMDB08097<br>HMDB07872<br>HMDB07873<br>HMDB07969<br>HMDB08097	C00157<br>C00157<br>C00157<br>C00157<br>C00157<br>C00157<br>C00157<br>C00157	Glycerophospholipids<br>Glycerophospholipids<br>Glycerophospholipids<br>Glycerophospholipids<br>Glycerophospholipids<br>Glycerophospholipids<br>Glycerophospholipids<br>Glycerophospholipids

PC aa C32:2	PC(14:0/18:2(9Z,12Z))<br>PC(16:1(9Z)/16:1(9Z))<br>PC(14:0/18:2(9Z,12Z))<br>PC(16:1(9Z)/16:1(9Z))	linoleoyl-sn-glycero-3-phosphocholine, GPCho(14:0/18:2), GPCho(14:0/18:2n6), GPCho(14:0/18:2w6), GPCho(32:2), Lecithin, PC aa C32:2, PC(14:0/18:2), PC(14:0/18:2n6), PC(14:0/18:2w6), PC(32:2), Phosphatidylcholine(14:0/18:2), Phosphatidylcholine(14:0/18:2n6), Phosphatidylcholine(14:0/18:2w6), Phosphatidylcholine(32:2)<br>1, 2-Dipalmitoleoyl-rac-glycero-3-phosphocholine, GPCho(16:1/16:1), GPCho(16:1n7/16:1n7), GPCho(16:1w7/16:1w7), GPCho(32:2), Lecithin, PC aa C32:2, PC(16:1/16:1), PC(16:1n7/16:1n7),	HMDB07874<br>HMDB08002<br>HMDB07874<br>HMDB08002	C00157<br>C00157<br>C00157<br>C00157	Glycerophospholipids<br>Glycerophospholipids<br>Glycerophospholipids<br>Glycerophospholipids

PC aa C32:3	PC(14:0/18:3(9Z,12Z,15Z))<br>PC(14:0/18:3(9Z,12Z,15Z))	linolenoyl-sn-glycero-3-phosphocholine, 1-Myristoyl-2-alpha-linolenoyl-sn-glycero-3-phosphocholine, GPCho(14:0/18:3), GPCho(14:0/18:3n3), GPCho(14:0/18:3w3), GPCho(32:3), Lecithin, PC aa C32:3, PC(14:0/18:3), PC(14:0/18:3n3), PC(14:0/18:3w3), PC(32:3), Phosphatidylcholine(14:0/18:3), Phosphatidylcholine(14:0/18:3n3), Phosphatidylcholine(14:0/18:3w3), Phosphatidylcholine(32:3)<br>1-Myristoyl-2-a-linolenoyl-sn-glycero-3-phosphocholine, 1-Myristoyl-2-alpha-linolenoyl-sn-glycero-3-phosphocholine, GPCho(14:0/18:3), GPCho(14:0/18:3n3), GPCho(14:0/18:3w3)	HMDB07876<br>HMDB07876	C00157<br>C00157	Glycerophospholipids<br>Glycerophospholipids

		vaccenoyl-sn-glycero-3-phosphocholine, GPCho(16:0/18:1), GPCho(16:0/18:1n7), GPCho(16:0/18:1w7), GPCho(34:1), Lecithin, PC aa C34:1, PC(16:0/18:1), PC(16:0/18:1n7), PC(16:0/18:1w7), PC(34:1), Phosphatidylcholine(16:0/18:1), Phosphatidylcholine(16:0/18:1n7), Phosphatidylcholine(16:0/18:1w7), Phosphatidylcholine(34:1)			
PC aa C34:1	PC(16:0/18:1(11Z))<br>PC(16:0/18:1(9Z))<br>PC(16:1(9Z)/18:0)<br>PC(18:0/16:1(9Z))<br>PC(18:1(9Z)/16:0)<br>PC(16:0/18:1(11Z))<br>PC(16:0/18:1(9Z))<br>PC(16:1(9Z)/18:0)<br>PC(18:0/16:1(9Z))<br>PC(18:1(9Z)/16:0)	1-Palmitoyl-2-oleoyl-sn-glycero-3-phosphocholine, GPCho(16:0/18:1), GPCho(16:0/18:1n9), GPCho(16:0/18:1w9), GPCho(34:1), Lecithin, PC aa C34:1, PC(16:0/18:1), PC(16:0/18:1n9), PC(16:0/18:1w9),	HMDB07971<br>HMDB07972<br>HMDB08003<br>HMDB08035<br>HMDB08100<br>HMDB07971<br>HMDB07972<br>HMDB08003<br>HMDB08035<br>HMDB08100	C00157<br>C00157<br>C00157<br>C00157<br>C00157<br>C00157<br>C00157<br>C00157<br>C00157<br>C00157	Glycerophospholipids<br>Glycerophospholipids<br>Glycerophospholipids<br>Glycerophospholipids<br>Glycerophospholipids<br>Glycerophospholipids<br>Glycerophospholipids<br>Glycerophospholipids<br>Glycerophospholipids<br>Glycerophospholipids

PC aa C34:2	PC(16:0/18:2(9Z,12Z))<br>PC(16:1(9Z)/18:1(11Z))<br>PC(16:1(9Z)/18:1(9Z))<br>PC(18:1(9Z)/16:1(9Z))<br>PC(18:2(9Z,12Z)/16:0)<br>PC(16:0/18:2(9Z,12Z))<br>PC(16:1(9Z)/18:1(11Z))<br>PC(16:1(9Z)/18:1(9Z))<br>PC(18:1(9Z)/16:1(9Z))<br>PC(18:2(9Z,12Z)/16:0)	linoleoyl-sn-glycero-3-phosphocholine, GPCho(16:0/18:2), GPCho(16:0/18:2n6), GPCho(16:0/18:2w6), GPCho(34:2), Lecithin, PC aa C34:2, PC(16:0/18:2), PC(16:0/18:2n6), PC(16:0/18:2w6), PC(34:2), Phosphatidylcholine(16:0/18:2), Phosphatidylcholine(16:0/18:2n6), Phosphatidylcholine(16:0/18:2w6), Phosphatidylcholine(34:2)<br>1-Palmitoleoyl-2-vaccenoyl-sn-glycero-3-phosphocholine, GPCho(16:1/18:1), GPCho(16:1n7/18:1n7), GPCho(16:1w7/18:1w7), GPCho(34:2), Lecithin, PC aa C34:2, PC(16:1/18:1), PC(16:1n7/18:1n7),	HMDB07973<br>HMDB08004<br>HMDB08005<br>HMDB08101<br>HMDB08133<br>HMDB07973<br>HMDB08004<br>HMDB08005<br>HMDB08101<br>HMDB08133	C00157<br>C00157<br>C00157<br>C00157<br>C00157<br>C00157<br>C00157<br>C00157<br>C00157<br>C00157	Glycerophospholipids<br>Glycerophospholipids<br>Glycerophospholipids<br>Glycerophospholipids<br>Glycerophospholipids<br>Glycerophospholipids<br>Glycerophospholipids<br>Glycerophospholipids<br>Glycerophospholipids<br>Glycerophospholipids

PC aa C34:3	PC(16:0/18:3(6Z,9Z,12Z))<br>PC(16:0/18:3(9Z,12Z,15Z))<br>PC(16:1(9Z)/18:2(9Z,12Z))<br>PC(16:0/18:3(6Z,9Z,12Z))<br>PC(16:0/18:3(9Z,12Z,15Z))<br>PC(16:1(9Z)/18:2(9Z,12Z))	linolenoyl-sn-glycero-3-phosphocholine, 1-Palmitoyl-2-gamma-linolenoyl-sn-glycero-3-phosphocholine, GPCho(16:0/18:3), GPCho(16:0/18:3n6), GPCho(16:0/18:3w6), GPCho(34:3), Lecithin, PC aa C34:3, PC(16:0/18:3), PC(16:0/18:3n6), PC(16:0/18:3w6), PC(34:3), Phosphatidylcholine(16:0/18:3), Phosphatidylcholine(16:0/18:3n6), Phosphatidylcholine(16:0/18:3w6), Phosphatidylcholine(34:3)<br>1-Palmitoyl-2-a-linolenoyl-sn-glycero-3-phosphocholine, 1-Palmitoyl-2-alpha-linolenoyl-sn-glycero-3-phosphocholine, GPCho(16:0/18:3), GPCho(16:0/18:3n3), GPCho(16:0/18:3w3)	HMDB07974<br>HMDB07975<br>HMDB08006<br>HMDB07974<br>HMDB07975<br>HMDB08006	C00157<br>C00157<br>C00157<br>C00157<br>C00157<br>C00157	Glycerophospholipids<br>Glycerophospholipids<br>Glycerophospholipids<br>Glycerophospholipids<br>Glycerophospholipids<br>Glycerophospholipids

		arachidonoyl-sn-glycero-3-phosphocholine, GPCho(14:0/20:4), GPCho(14:0/20:4n6), GPCho(14:0/20:4w6), GPCho(34:4), Lecithin, PC aa C34:4, PC(14:0/20:4), PC(14:0/20:4n6), PC(14:0/20:4w6), PC(34:4), Phosphatidylcholine(14:0/20:4), Phosphatidylcholine(14:0/20:4n6), Phosphatidylcholine(14:0/20:4w6), Phosphatidylcholine(34:4)			
PC aa C34:4	PC(14:0/20:4(5Z,8Z,11Z,14Z)) PC(16:0/18:4(6Z,9Z,12Z,15Z)) PC(14:0/20:4(5Z,8Z,11Z,14Z)) PC(16:0/18:4(6Z,9Z,12Z,15Z))	1-Palmitoyl-2-stearidonoyl-sn-glycero-3-phosphocholine, GPCho(16:0/18:4), GPCho(16:0/18:4n3), GPCho(16:0/18:4w3), GPCho(34:4), Lecithin, PC aa C34:4, PC(16:0/18:4),	HMDB07883 HMDB07976 HMDB07883 HMDB07976	C00157 C00157 C00157 C00157	Glycerophospholipids Glycerophospholipids Glycerophospholipids Glycerophospholipids

PC aa C36:0	PC(14:0/22:0)<br>PC(16:0/20:0)<br>PC(18:0/18:0)<br>PC(20:0/16:0)<br>PC(22:0/14:0)<br>PC(14:0/22:0)<br>PC(16:0/20:0)<br>PC(18:0/18:0)<br>PC(20:0/16:0)<br>PC(22:0/14:0)	behenoyl-sn-glycero-3-phosphocholine, GPCho(14:0/22:0), GPCho(36:0), Lecithin, PC aa C36:0, PC(14:0/22:0), PC(36:0), Phosphatidylcholine(14:0/22:0), Phosphatidylcholine(36:0)<br>1-Palmitoyl-2-arachidonyl-sn-glycero-3-phosphocholine, GPCho(16:0/20:0), GPCho(36:0), Lecithin, PC aa C36:0, PC(16:0/20:0), PC(36:0), Phosphatidylcholine(16:0/20:0), Phosphatidylcholine(36:0)<br>1, 2-Distearoyl-rac-glycero-3-phosphocholine, GPCho(18:0/18:0), GPCho(36:0), Lecithin, PC aa	HMDB07886<br>HMDB07977<br>HMDB08036<br>HMDB08265<br>HMDB08525<br>HMDB07886<br>HMDB07977<br>HMDB08036<br>HMDB08265<br>HMDB08525	C00157<br>C00157<br>C00157<br>C00157<br>C00157<br>C00157<br>C00157<br>C00157<br>C00157<br>C00157	Glycerophospholipids<br>Glycerophospholipids<br>Glycerophospholipids<br>Glycerophospholipids<br>Glycerophospholipids<br>Glycerophospholipids<br>Glycerophospholipids<br>Glycerophospholipids<br>Glycerophospholipids<br>Glycerophospholipids

		vaccenoyl-sn-glycero-3-phosphocholine, GPCho(18:0/18:1), GPCho(18:0/18:1n7), GPCho(18:0/18:1w7), GPCho(36:1), Lecithin, PC aa C36:1, PC(18:0/18:1), PC(18:0/18:1n7), PC(18:0/18:1w7), PC(36:1), Phosphatidylcholine(18:0/18:1), Phosphatidylcholine(18:0/18:1n7), Phosphatidylcholine(18:0/18:1w7), Phosphatidylcholine(36:1)			
PC aa C36:1	PC(18:0/18:1(11Z))<br>PC(18:0/18:1(9Z))<br>PC(18:1(11Z)/18:0)<br>PC(18:1(9Z)/18:0)<br>PC(18:0/18:1(11Z))<br>PC(18:0/18:1(9Z))<br>PC(18:1(11Z)/18:0)<br>PC(18:1(9Z)/18:0)	1-Stearoyl-2-oleoyl-sn-glycero-3-phosphocholine, GPCho(18:0/18:1), GPCho(18:0/18:1n9), GPCho(18:0/18:1w9), GPCho(36:1), Lecithin, PC aa C36:1, PC(18:0/18:1), PC(18:0/18:1n9), PC(18:0/18:1w9),	HMDB08037<br>HMDB08038<br>HMDB08069<br>HMDB08102<br>HMDB08037<br>HMDB08038<br>HMDB08069<br>HMDB08102	C00157<br>C00157<br>C00157<br>C00157<br>C00157<br>C00157<br>C00157<br>C00157	Glycerophospholipids<br>Glycerophospholipids<br>Glycerophospholipids<br>Glycerophospholipids<br>Glycerophospholipids<br>Glycerophospholipids<br>Glycerophospholipids<br>Glycerophospholipids

PC aa C36:2	PC(18:0/18:2(9Z,12Z)) PC(18:1(11Z)/18:1(11Z)) PC(18:2(9Z,12Z)/18:0) PC(18:1(9Z)/18:1(9Z)) PC(18:0/18:2(9Z,12Z)) PC(18:1(11Z)/18:1(11Z)) PC(18:2(9Z,12Z)/18:0)	sn-glycero-3-phosphocholine, GPCho(18:0/18:2), GPCho(18:0/18:2n6), GPCho(18:0/18:2w6), GPCho(36:2), Lecithin, PC aa C36:2, PC(18:0/18:2), PC(18:0/18:2n6), PC(18:0/18:2w6), PC(36:2), Phosphatidylcholine(18:0/18:2), Phosphatidylcholine(18:0/18:2n6), Phosphatidylcholine(18:0/18:2w6), Phosphatidylcholine(36:2) 1, 2-Divaccenoyl-rac-glycero-3-phosphocholine, GPCho(18:1/18:1), GPCho(18:1n7/18:1n7), GPCho(18:1w7/18:1w7), GPCho(36:2), Lecithin, PC aa C36:2, PC(18:1/18:1), PC(18:1n7/18:1n7),	HMDB08039 HMDB08070 HMDB08135 HMDB00593 HMDB08039 HMDB08070 HMDB08135	C00157 C00157 C00157 C00157 C00157 C00157 C00157	Glycerophospholipids Glycerophospholipids Glycerophospholipids Glycerophospholipids Glycerophospholipids Glycerophospholipids Glycerophospholipids

PC aa C36:3	PC(16:0/20:3(5Z,8Z,11Z))<br>PC(16:0/20:3(8Z,11Z,14Z))<br>PC(18:0/18:3(6Z,9Z,12Z))<br>PC(18:1(9Z)/18:2(9Z,12Z))<br>PC(16:0/20:3(5Z,8Z,11Z))<br>PC(16:0/20:3(8Z,11Z,14Z))<br>PC(18:0/18:3(6Z,9Z,12Z))<br>PC(18:1(9Z)/18:2(9Z,12Z))	meadoyl-sn-glycero-3-phosphocholine, GPCho(16:0/20:3), GPCho(16:0/20:3n9), GPCho(16:0/20:3w9), GPCho(36:3), Lecithin, PC aa C36:3, PC(16:0/20:3), PC(16:0/20:3n9), PC(16:0/20:3w9), PC(36:3), Phosphatidylcholine(16:0/20:3), Phosphatidylcholine(16:0/20:3n9), Phosphatidylcholine(16:0/20:3w9), Phosphatidylcholine(36:3)<br>1-Palmitoyl-2-homo-g-linolenoyl-sn-glycero-3-phosphocholine, 1-Palmitoyl-2-homo-gamma-linolenoyl-sn-glycero-3-phosphocholine, GPCho(16:0/20:3), GPCho(16:0/20:3n6), GPCho(16:0/20:3w6), GPCho(36:3),	HMDB07980<br>HMDB07981<br>HMDB08040<br>HMDB08105<br>HMDB07980<br>HMDB07981<br>HMDB08040<br>HMDB08105	C00157<br>C00157<br>C00157<br>C00157<br>C00157<br>C00157<br>C00157<br>C00157	Glycerophospholipids<br>Glycerophospholipids<br>Glycerophospholipids<br>Glycerophospholipids<br>Glycerophospholipids<br>Glycerophospholipids<br>Glycerophospholipids<br>Glycerophospholipids

	PC(16:0/20:4(5Z,8Z,	arachidonoyl-sn-			
	11Z,14Z))	glycero-3-			
	PC(18:0/18:4(6Z,9Z,	phosphocholine,			
	12Z,15Z))	GPCho(16:0/20:4),			
	PC(18:1(9Z)/18:3(6Z,	GPCho(16:0/20:4n6),			
	9Z,12Z))	GPCho(16:0/20:4w6)			
	PC(18:1(9Z)/18:3(9Z,	, GPCho(36:4),			
	12Z,15Z))	Lecithin, PC aa			
	PC(18:2(9Z,12Z)/18:	C36:4,			
	2(9Z,12Z))	PC(16:0/20:4),			
	PC(18:3(6Z,9Z,12Z)/	PC(16:0/20:4n6),			
	18:1(9Z))	PC(16:0/20:4w6),			
	PC(18:3(9Z,12Z,15Z)	PC(36:4),			
	/18:1(9Z))	Phosphatidylcholine(			
	PC(20:4(5Z,8Z,11Z,1	16:0/20:4),			
	4Z)/16:0)	Phosphatidylcholine(			
	PC(16:0/20:4(5Z,8Z,	16:0/20:4n6),	HMDB07982	C00157	Glycerophospholipids
	11Z,14Z))	Phosphatidylcholine(	HMDB08042	C00157	Glycerophospholipids
	PC(18:0/18:4(6Z,9Z,	16:0/20:4w6),	HMDB08106	C00157	Glycerophospholipids
	12Z,15Z))	Phosphatidylcholine(	HMDB08107	C00157	Glycerophospholipids
	PC(18:1(9Z)/18:3(6Z,	36:4)	HMDB08138	C00157	Glycerophospholipids
	9Z,12Z))	1-Stearoyl-2-	HMDB08170	C00157	Glycerophospholipids
	PC(18:1(9Z)/18:3(9Z,	stearidonoyl-sn-	HMDB08203	C00157	Glycerophospholipids
	12Z,15Z))	glycero-3-	HMDB08429	C00157	Glycerophospholipids
	PC(18:2(9Z,12Z)/18:	phosphocholine,	HMDB07982	C00157	Glycerophospholipids
	2(9Z,12Z))	GPCho(18:0/18:4),	HMDB08042	C00157	Glycerophospholipids
	PC(18:3(6Z,9Z,12Z)/	GPCho(18:0/18:4n3),	HMDB08106	C00157	Glycerophospholipids
	18:1(9Z))	GPCho(18:0/18:4w3)	HMDB08107	C00157	Glycerophospholipids
	PC(18:3(9Z,12Z,15Z)	, GPCho(36:4),	HMDB08138	C00157	Glycerophospholipids
	/18:1(9Z))	Lecithin, PC aa	HMDB08170	C00157	Glycerophospholipids
	PC(20:4(5Z,8Z,11Z,1	C36:4,	HMDB08203	C00157	Glycerophospholipids
PC aa C36:4	4Z)/16:0)	PC(18:0/18:4),	HMDB08429	C00157	Glycerophospholipids

		eicosapentaenoyl-sn-glycero-3-phosphocholine, GPCho(16:0/20:5), GPCho(16:0/20:5n3), GPCho(16:0/20:5w3), GPCho(36:5), Lecithin, PC aa C36:5, PC(16:0/20:5), PC(16:0/20:5n3), PC(16:0/20:5w3), PC(36:5), Phosphatidylcholine(16:0/20:5), Phosphatidylcholine(16:0/20:5n3), Phosphatidylcholine(16:0/20:5w3), Phosphatidylcholine(36:5)			
PC aa C36:5	PC(16:0/20:5(5Z,8Z,11Z,14Z,17Z))<br>PC(16:1(9Z)/20:4(5Z,8Z,11Z,14Z))<br>PC(16:0/20:5(5Z,8Z,11Z,14Z,17Z))<br>PC(16:1(9Z)/20:4(5Z,8Z,11Z,14Z))	1-Palmitoleoyl-2-arachidonoyl-sn-glycero-3-phosphocholine, GPCho(16:1/20:4), GPCho(16:1n7/20:4n6), GPCho(16:1w7/20:4w6), GPCho(36:5), Lecithin, PC aa C36:5,	HMDB07984<br>HMDB08015<br>HMDB07984<br>HMDB08015	C00157<br>C00157<br>C00157<br>C00157	Glycerophospholipids<br>Glycerophospholipids<br>Glycerophospholipids<br>Glycerophospholipids

PC aa C36:6	PC(14:0/22:6(4Z,7Z,10Z,13Z,16Z,19Z))<br>PC(18:3(9Z,12Z,15Z)/18:3(9Z,12Z,15Z))<br>PC(14:0/22:6(4Z,7Z,10Z,13Z,16Z,19Z))<br>PC(18:3(9Z,12Z,15Z)/18:3(9Z,12Z,15Z))	docosahexaenoyl-sn-glycero-3-phosphocholine, GPCho(14:0/22:6), GPCho(14:0/22:6n3), GPCho(14:0/22:6w3), GPCho(36:6), Lecithin, PC aa C36:6, PC(14:0/22:6), PC(14:0/22:6n3), PC(14:0/22:6w3), PC(36:6), Phosphatidylcholine(14:0/22:6), Phosphatidylcholine(14:0/22:6n3), Phosphatidylcholine(14:0/22:6w3), Phosphatidylcholine(36:6)<br>1, 2-Dia-linolenoyl-rac-glycero-3-phosphocholine, GPCho(18:3/18:3), GPCho(18:3n3/18:3n3), GPCho(18:3w3/18:3w3), GPCho(36:6), Lecithin, PC aa C36:6, PC(18:3/18:3),	HMDB07892<br>HMDB08206<br>HMDB07892<br>HMDB08206	C00157<br>C00157<br>C00157<br>C00157	Glycerophospholipids<br>Glycerophospholipids<br>Glycerophospholipids<br>Glycerophospholipids

PC aa C38:0	PC(14:0/24:0)<br>PC(16:0/22:0)<br>PC(18:0/20:0)<br>PC(20:0/18:0)<br>PC(22:0/16:0)<br>PC(24:0/14:0)<br>PC(14:0/24:0)<br>PC(16:0/22:0)<br>PC(18:0/20:0)<br>PC(20:0/18:0)<br>PC(22:0/16:0)<br>PC(24:0/14:0)	lignoceroyl-sn-<br>glycero-3-<br>phosphocholine,<br>GPCho(14:0/24:0),<br>GPCho(38:0),<br>Lecithin, PC aa<br>C38:0,<br>PC(14:0/24:0),<br>PC(38:0),<br>Phosphatidylcholine(<br>14:0/24:0),<br>Phosphatidylcholine(<br>38:0)<br>1-Palmitoyl-2-<br>behenoyl-sn-glycero-<br>3-phosphocholine,<br>GPCho(16:0/22:0),<br>GPCho(38:0),<br>Lecithin, PC aa<br>C38:0,<br>PC(16:0/22:0),<br>PC(38:0),<br>Phosphatidylcholine(<br>16:0/22:0),<br>Phosphatidylcholine(<br>38:0)<br>1-Stearoyl-2-<br>arachidonyl-sn-<br>glycero-3-<br>phosphocholine,<br>GPCho(18:0/20:0),<br>GPCho(38:0),	HMDB07893<br>HMDB07985<br>HMDB08043<br>HMDB08267<br>HMDB08528<br>HMDB08755<br>HMDB07893<br>HMDB07985<br>HMDB08043<br>HMDB08267<br>HMDB08528<br>HMDB08755	C00157<br>C00157<br>C00157<br>C00157<br>C00157<br>C00157<br>C00157<br>C00157<br>C00157<br>C00157<br>C00157<br>C00157	Glycerophospholipids<br>Glycerophospholipids<br>Glycerophospholipids<br>Glycerophospholipids<br>Glycerophospholipids<br>Glycerophospholipids<br>Glycerophospholipids<br>Glycerophospholipids<br>Glycerophospholipids<br>Glycerophospholipids<br>Glycerophospholipids<br>Glycerophospholipids

		nervonoyl-sn-glycero-3-phosphocholine, GPCho(14:0/24:1), GPCho(14:0/24:1n9), GPCho(14:0/24:1w9), GPCho(38:1), Lecithin, PC aa C38:1, PC(14:0/24:1), PC(14:0/24:1n9), PC(14:0/24:1w9), PC(38:1), Phosphatidylcholine(14:0/24:1), Phosphatidylcholine(14:0/24:1n9), Phosphatidylcholine(14:0/24:1w9), Phosphatidylcholine(38:1)			
	PC(14:0/24:1(15Z))	1-Palmitoyl-2-erucoyl-	HMDB07894	C00157	Glycerophospholipids
	PC(16:0/22:1(13Z))	sn-glycero-3-	HMDB07986	C00157	Glycerophospholipids
	PC(18:0/20:1(11Z))	phosphocholine,	HMDB08044	C00157	Glycerophospholipids
	PC(18:1(9Z)/20:0)	GPCho(16:0/22:1),	HMDB08109	C00157	Glycerophospholipids
	PC(20:0/18:1(11Z))	GPCho(16:0/22:1n9),	HMDB08268	C00157	Glycerophospholipids
	PC(20:0/18:1(9Z))	GPCho(16:0/22:1w9)	HMDB08269	C00157	Glycerophospholipids
	PC(14:0/24:1(15Z))	, GPCho(38:1),	HMDB07894	C00157	Glycerophospholipids
	PC(16:0/22:1(13Z))	Lecithin, PC aa	HMDB07986	C00157	Glycerophospholipids
	PC(18:0/20:1(11Z))	C38:1,	HMDB08044	C00157	Glycerophospholipids
	PC(18:1(9Z)/20:0)	PC(16:0/22:1),	HMDB08109	C00157	Glycerophospholipids
	PC(20:0/18:1(11Z))	PC(16:0/22:1n9),	HMDB08268	C00157	Glycerophospholipids
PC aa C38:1	PC(20:0/18:1(9Z))	PC(16:0/22:1w9),	HMDB08269	C00157	Glycerophospholipids

PC aa C38:3	PC(18:0/20:3(5Z,8Z,11Z)) PC(18:0/20:3(8Z,11Z,14Z)) PC(18:0/20:3(5Z,8Z,11Z)) PC(18:0/20:3(8Z,11Z,14Z))	sn-glycero-3-phosphocholine, GPCho(18:0/20:3), GPCho(18:0/20:3n9), GPCho(18:0/20:3w9), GPCho(38:3), Lecithin, PC aa C38:3, PC(18:0/20:3), PC(18:0/20:3n9), PC(18:0/20:3w9), PC(38:3), Phosphatidylcholine(18:0/20:3), Phosphatidylcholine(18:0/20:3n9), Phosphatidylcholine(18:0/20:3w9), Phosphatidylcholine(38:3) 1-Stearoyl-2-homo-g-linolenoyl-sn-glycero-3-phosphocholine, 1-Stearoyl-2-homo-gamma-linolenoyl-sn-glycero-3-phosphocholine, GPCho(18:0/20:3), GPCho(18:0/20:3n6), GPCho(18:0/20:3w6), GPCho(38:3), Lecithin, PC aa	HMDB08046 HMDB08047 HMDB08046 HMDB08047	C00157 C00157 C00157 C00157	Glycerophospholipids Glycerophospholipids Glycerophospholipids Glycerophospholipids

PC aa C38:4	PC(16:0/22:4(7Z,10Z,13Z,16Z))<br>PC(18:0/20:4(5Z,8Z,11Z,14Z))<br>PC(18:1(9Z)/20:3(5Z,8Z,11Z))<br>PC(16:0/22:4(7Z,10Z,13Z,16Z))<br>PC(18:0/20:4(5Z,8Z,11Z,14Z))<br>PC(18:1(9Z)/20:3(5Z,8Z,11Z))	adrenoyl-sn-glycero-3-phosphocholine, GPCho(16:0/22:4), GPCho(16:0/22:4n6), GPCho(16:0/22:4w6), GPCho(38:4), Lecithin, PC aa C38:4, PC(16:0/22:4), PC(16:0/22:4n6), PC(16:0/22:4w6), PC(38:4), Phosphatidylcholine(16:0/22:4), Phosphatidylcholine(16:0/22:4n6), Phosphatidylcholine(16:0/22:4w6), Phosphatidylcholine(38:4)<br>1-Stearoyl-2-arachidonoyl-sn-glycero-3-phosphocholine, GPCho(18:0/20:4), GPCho(18:0/20:4n6), GPCho(18:0/20:4w6), GPCho(38:4), Lecithin, PC aa C38:4, PC(18:0/20:4), PC(18:0/20:4n6),	HMDB07988<br>HMDB08048<br>HMDB08112<br>HMDB07988<br>HMDB08048<br>HMDB08112	C00157<br>C00157<br>C00157<br>C00157<br>C00157<br>C00157	Glycerophospholipids<br>Glycerophospholipids<br>Glycerophospholipids<br>Glycerophospholipids<br>Glycerophospholipids<br>Glycerophospholipids

PC aa C38:5	PC(16:0/22:5(4Z,7Z,<br>10Z,13Z,16Z))<br>PC(16:0/22:5(7Z,10Z<br>,13Z,16Z,19Z))<br>PC(18:0/20:5(5Z,8Z,<br>11Z,14Z,17Z))<br>PC(18:1(9Z)/20:4(5Z,<br>8Z,11Z,14Z))<br>PC(16:0/22:5(4Z,7Z,<br>10Z,13Z,16Z))<br>PC(16:0/22:5(7Z,10Z<br>,13Z,16Z,19Z))<br>PC(18:0/20:5(5Z,8Z,<br>11Z,14Z,17Z))<br>PC(18:1(9Z)/20:4(5Z,<br>8Z,11Z,14Z))	docosapentaenoyl-sn-<br>glycero-3-<br>phosphocholine, 1-<br>Palmitoyl-2-<br>osbondoyl-sn-glycero-<br>3-phosphocholine,<br>GPCho(16:0/22:5),<br>GPCho(16:0/22:5n6),<br>GPCho(16:0/22:5w6)<br>, GPCho(38:5),<br>Lecithin, PC aa<br>C38:5,<br>PC(16:0/22:5),<br>PC(16:0/22:5n6),<br>PC(16:0/22:5w6),<br>PC(38:5),<br>Phosphatidylcholine(<br>16:0/22:5),<br>Phosphatidylcholine(<br>16:0/22:5n6),<br>Phosphatidylcholine(<br>16:0/22:5w6),<br>Phosphatidylcholine(<br>38:5)<br>1-Palmitoyl-2-<br>docosapentaenoyl-sn-<br>glycero-3-<br>phosphocholine,<br>GPCho(16:0/22:5),<br>GPCho(16:0/22:5n3),<br>GPCho(16:0/22:5w3)<br>, GPCho(38:5),	HMDB07989<br>HMDB07990<br>HMDB08050<br>HMDB08114<br>HMDB07989<br>HMDB07990<br>HMDB08050<br>HMDB08114	C00157<br>C00157<br>C00157<br>C00157<br>C00157<br>C00157<br>C00157<br>C00157	Glycerophospholipids<br>Glycerophospholipids<br>Glycerophospholipids<br>Glycerophospholipids<br>Glycerophospholipids<br>Glycerophospholipids<br>Glycerophospholipids<br>Glycerophospholipids

PC aa C38:6	PC(16:0/22:6(4Z,7Z,10Z,13Z,16Z,19Z))<br>PC(18:1(11Z)/20:5(5Z,8Z,11Z,14Z,17Z))<br>PC(18:1(9Z)/20:5(5Z,8Z,11Z,14Z,17Z))<br>PC(18:2(9Z,12Z)/20:4(5Z,8Z,11Z,14Z))<br>PC(20:4(5Z,8Z,11Z,14Z)/18:2(9Z,12Z))<br>PC(20:5(5Z,8Z,11Z,14Z,17Z)/18:1(9Z))<br>PC(22:6(4Z,7Z,10Z,13Z,16Z,19Z)/16:0)<br>PC(16:0/22:6(4Z,7Z,10Z,13Z,16Z,19Z))<br>PC(18:1(11Z)/20:5(5Z,8Z,11Z,14Z,17Z))<br>PC(18:1(9Z)/20:5(5Z,8Z,11Z,14Z,17Z))<br>PC(18:2(9Z,12Z)/20:4(5Z,8Z,11Z,14Z))<br>PC(20:4(5Z,8Z,11Z,14Z)/18:2(9Z,12Z))<br>PC(20:5(5Z,8Z,11Z,14Z,17Z)/18:1(9Z))<br>PC(22:6(4Z,7Z,10Z,13Z,16Z,19Z)/16:0)	docosahexaenoyl-sn-glycero-3-phosphocholine, GPCho(16:0/22:6), GPCho(16:0/22:6n3), GPCho(16:0/22:6w3), GPCho(38:6), Lecithin, PC aa C38:6, PC(16:0/22:6), PC(16:0/22:6n3), PC(16:0/22:6w3), PC(38:6), Phosphatidylcholine(16:0/22:6), Phosphatidylcholine(16:0/22:6n3), Phosphatidylcholine(16:0/22:6w3), Phosphatidylcholine(38:6) 1-Vaccenoyl-2-eicosapentaenoyl-sn-glycero-3-phosphocholine, GPCho(18:1/20:5), GPCho(18:1n7/20:5n3), GPCho(18:1w7/20:5w3), GPCho(38:6), Lecithin, PC aa C38:6,	HMDB07991<br>HMDB08083<br>HMDB08116<br>HMDB08147<br>HMDB08434<br>HMDB08499<br>HMDB08725<br>HMDB07991<br>HMDB08083<br>HMDB08116<br>HMDB08147<br>HMDB08434<br>HMDB08499<br>HMDB08725	C00157<br>C00157<br>C00157<br>C00157<br>C00157<br>C00157<br>C00157<br>C00157<br>C00157<br>C00157<br>C00157<br>C00157<br>C00157<br>C00157	Glycerophospholipids<br>Glycerophospholipids<br>Glycerophospholipids<br>Glycerophospholipids<br>Glycerophospholipids<br>Glycerophospholipids<br>Glycerophospholipids<br>Glycerophospholipids<br>Glycerophospholipids<br>Glycerophospholipids<br>Glycerophospholipids<br>Glycerophospholipids<br>Glycerophospholipids<br>Glycerophospholipids

		nervonoyl-sn-glycero-			
		3-phosphocholine,			
		GPCho(16:0/24:1),			
		GPCho(16:0/24:1n9),			
		GPCho(16:0/24:1w9)			
		, GPCho(40:1),			
		Lecithin, PC aa			
		C40:1,			
		PC(16:0/24:1),			
		PC(16:0/24:1n9),			
		PC(16:0/24:1w9),			
		PC(40:1),			
		Phosphatidylcholine(			
		16:0/24:1),			
		Phosphatidylcholine(			
		16:0/24:1n9),			
		Phosphatidylcholine(			
		16:0/24:1w9),			
		Phosphatidylcholine(			
		40:1)			
		1-Stearoyl-2-erucoyl-			
		sn-glycero-3-			
	PC(16:0/24:1(15Z))	phosphocholine,	HMDB07993	C00157	Glycerophospholipids
	PC(18:0/22:1(13Z))	GPCho(18:0/22:1),	HMDB08052	C00157	Glycerophospholipids
	PC(18:1(11Z)/22:0)	GPCho(18:0/22:1n9),	HMDB08084	C00157	Glycerophospholipids
	PC(18:1(9Z)/22:0)	GPCho(18:0/22:1w9)	HMDB08117	C00157	Glycerophospholipids
	PC(20:0/20:1(11Z))	, GPCho(40:1),	HMDB08275	C00157	Glycerophospholipids
	PC(16:0/24:1(15Z))	Lecithin, PC aa	HMDB07993	C00157	Glycerophospholipids
	PC(18:0/22:1(13Z))	C40:1,	HMDB08052	C00157	Glycerophospholipids
	PC(18:1(11Z)/22:0)	PC(18:0/22:1),	HMDB08084	C00157	Glycerophospholipids
	PC(18:1(9Z)/22:0)	PC(18:0/22:1n9),	HMDB08117	C00157	Glycerophospholipids
PC aa C40:1	PC(20:0/20:1(11Z))	PC(18:0/22:1w9),	HMDB08275	C00157	Glycerophospholipids

		eicosadienoyl-sn-glycero-3-phosphocholine, GPCho(20:0/20:2), GPCho(20:0/20:2n6), GPCho(20:0/20:2w6), GPCho(40:2), Lecithin, PC aa C40:2, PC(20:0/20:2), PC(20:0/20:2n6), PC(20:0/20:2w6), PC(40:2), Phosphatidylcholine(20:0/20:2), Phosphatidylcholine(20:0/20:2n6), Phosphatidylcholine(20:0/20:2w6), Phosphatidylcholine(40:2)			
PC aa C40:2	PC(20:0/20:2(11Z,14Z)) PC(20:1(11Z)/20:1(11Z)) PC(20:0/20:2(11Z,14Z)) PC(20:1(11Z)/20:1(11Z))	1, 2-Dieicosenoyl-rac-glycero-3-phosphocholine, GPCho(20:1/20:1), GPCho(20:1n9/20:1n9), GPCho(20:1w9/20:1w9), GPCho(40:2), Lecithin, PC aa C40:2, PC(20:1/20:1),	HMDB08276 HMDB08308 HMDB08276 HMDB08308	C00157 C00157 C00157 C00157	Glycerophospholipids Glycerophospholipids Glycerophospholipids Glycerophospholipids
PC aa C40:3					

		adrenoyl-sn-glycero-3-phosphocholine, GPCho(18:0/22:4), GPCho(18:0/22:4n6), GPCho(18:0/22:4w6), GPCho(40:4), Lecithin, PC aa C40:4, PC(18:0/22:4), PC(18:0/22:4n6), PC(18:0/22:4w6), PC(40:4), Phosphatidylcholine(18:0/22:4), Phosphatidylcholine(18:0/22:4n6), Phosphatidylcholine(18:0/22:4w6), Phosphatidylcholine(40:4)			
PC aa C40:4	PC(18:0/22:4(7Z,10Z,13Z,16Z))<br>PC(20:0/20:4(5Z,8Z,11Z,14Z))<br>PC(22:4(7Z,10Z,13Z,16Z)/18:0)<br>PC(18:0/22:4(7Z,10Z,13Z,16Z))<br>PC(20:0/20:4(5Z,8Z,11Z,14Z))<br>PC(22:4(7Z,10Z,13Z,16Z)/18:0)	1-Arachidonyl-2-arachidonoyl-sn-glycero-3-phosphocholine, GPCho(20:0/20:4), GPCho(20:0/20:4n6), GPCho(20:0/20:4w6), GPCho(40:4), Lecithin, PC aa C40:4, PC(20:0/20:4), PC(20:0/20:4n6),	HMDB08054<br>HMDB08279<br>HMDB08628<br>HMDB08054<br>HMDB08279<br>HMDB08628	C00157<br>C00157<br>C00157<br>C00157<br>C00157<br>C00157	Glycerophospholipids<br>Glycerophospholipids<br>Glycerophospholipids<br>Glycerophospholipids<br>Glycerophospholipids<br>Glycerophospholipids

PC aa C40:5	PC(18:0/22:5(4Z,7Z, 10Z,13Z,16Z)) PC(18:0/22:5(7Z,10Z ,13Z,16Z,19Z)) PC(18:1(9Z)/22:4(7Z, 10Z,13Z,16Z)) PC(18:0/22:5(4Z,7Z, 10Z,13Z,16Z)) PC(18:0/22:5(7Z,10Z ,13Z,16Z,19Z)) PC(18:1(9Z)/22:4(7Z, 10Z,13Z,16Z))	docosapentaenoyl-sn-glycero-3-phosphocholine, 1-Stearoyl-2-osbondoyl-sn-glycero-3-phosphocholine, GPCho(18:0/22:5), GPCho(18:0/22:5n6), GPCho(18:0/22:5w6) , GPCho(40:5), Lecithin, PC(18:0/22:5), PC(18:0/22:5n6), PC(18:0/22:5w6), PC(40:5), Phosphatidylcholine(18:0/22:5), Phosphatidylcholine(18:0/22:5n6), Phosphatidylcholine(18:0/22:5w6), Phosphatidylcholine(40:5) 1-Stearoyl-2-docosapentaenoyl-sn-glycero-3-phosphocholine, GPCho(18:0/22:5), GPCho(18:0/22:5n3), GPCho(18:0/22:5w3) , GPCho(40:5), Lecithin,	HMDB08055 HMDB08056 HMDB08120 HMDB08055 HMDB08056 HMDB08120	C00157 C00157 C00157 C00157 C00157 C00157	Glycerophospholipids Glycerophospholipids Glycerophospholipids Glycerophospholipids Glycerophospholipids Glycerophospholipids

PC aa C40:6	PC(18:0/22:6(4Z,7Z,10Z,13Z,16Z,19Z))<br>PC(18:1(11Z)/22:5(7Z,10Z,13Z,16Z,19Z))<br>PC(18:1(9Z)/22:5(7Z,10Z,13Z,16Z,19Z))<br>PC(18:0/22:6(4Z,7Z,10Z,13Z,16Z,19Z))<br>PC(18:1(11Z)/22:5(7Z,10Z,13Z,16Z,19Z))<br>PC(18:1(9Z)/22:5(7Z,10Z,13Z,16Z,19Z))	docosahexaenoyl-sn-glycero-3-phosphocholine, GPCho(18:0/22:6), GPCho(18:0/22:6n3), GPCho(18:0/22:6w3), GPCho(40:6), Lecithin, PC aa C40:6, PC(18:0/22:6), PC(18:0/22:6n3), PC(18:0/22:6w3), PC(40:6), Phosphatidylcholine(18:0/22:6), Phosphatidylcholine(18:0/22:6n3), Phosphatidylcholine(18:0/22:6w3), Phosphatidylcholine(40:6)<br>1-Vaccenoyl-2-docosapentaenoyl-sn-glycero-3-phosphocholine, GPCho(18:1/22:5), GPCho(18:1n7/22:5n3), GPCho(18:1w7/22:5w3), GPCho(40:6), Lecithin, PC aa C40:6,	HMDB08057<br>HMDB08089<br>HMDB08122<br>HMDB08057<br>HMDB08089<br>HMDB08122	C00157<br>C00157<br>C00157<br>C00157<br>C00157<br>C00157	Glycerophospholipids<br>Glycerophospholipids<br>Glycerophospholipids<br>Glycerophospholipids<br>Glycerophospholipids<br>Glycerophospholipids

PC aa C42:0	PC(18:0/24:0)<br>PC(20:0/22:0)<br>PC(22:0/20:0)<br>PC(24:0/18:0)<br>PC(18:0/24:0)<br>PC(20:0/22:0)<br>PC(22:0/20:0)<br>PC(24:0/18:0)	lignoceroyl-sn-glycero-3-phosphocholine, GPCho(18:0/24:0), GPCho(42:0), Lecithin, PC aa C42:0, PC(18:0/24:0), PC(42:0), Phosphatidylcholine(18:0/24:0), Phosphatidylcholine(42:0)<br>1-Arachidonyl-2-behenoyl-sn-glycero-3-phosphocholine, GPCho(20:0/22:0), GPCho(42:0), Lecithin, PC aa C42:0, PC(20:0/22:0), PC(42:0), Phosphatidylcholine(20:0/22:0), Phosphatidylcholine(42:0)<br>1-Behenoyl-2-arachidonyl-sn-glycero-3-phosphocholine, GPCho(22:0/20:0), GPCho(42:0),	HMDB08058<br>HMDB08282<br>HMDB08537<br>HMDB08760<br>HMDB08058<br>HMDB08282<br>HMDB08537<br>HMDB08760	C00157<br>C00157<br>C00157<br>C00157<br>C00157<br>C00157<br>C00157<br>C00157	Glycerophospholipids<br>Glycerophospholipids<br>Glycerophospholipids<br>Glycerophospholipids<br>Glycerophospholipids<br>Glycerophospholipids<br>Glycerophospholipids<br>Glycerophospholipids

PC aa C42:1	PC(18:0/24:1(15Z))<br>PC(18:1(9Z)/24:0)<br>PC(20:0/22:1(13Z))<br>PC(22:0/20:1(11Z))<br>PC(24:0/18:1(9Z))<br>PC(18:0/24:1(15Z))<br>PC(18:1(9Z)/24:0)<br>PC(20:0/22:1(13Z))<br>PC(22:0/20:1(11Z))<br>PC(24:0/18:1(9Z))	nervonoyl-sn-glycero-3-phosphocholine, GPCho(18:0/24:1), GPCho(18:0/24:1n9), GPCho(18:0/24:1w9), GPCho(42:1), Lecithin, PC aa C42:1, PC(18:0/24:1), PC(18:0/24:1n9), PC(18:0/24:1w9), PC(42:1), Phosphatidylcholine(18:0/24:1), Phosphatidylcholine(18:0/24:1n9), Phosphatidylcholine(18:0/24:1w9), Phosphatidylcholine(42:1)<br>1-Oleoyl-2-lignoceroyl-sn-glycero-3-phosphocholine, GPCho(18:1/24:0), GPCho(18:1n9/24:0), GPCho(18:1w9/24:0), GPCho(42:1), Lecithin, PC aa C42:1, PC(18:1/24:0), PC(18:1n9/24:0),	HMDB08059<br>HMDB08124<br>HMDB08283<br>HMDB08538<br>HMDB08762<br>HMDB08059<br>HMDB08124<br>HMDB08283<br>HMDB08538<br>HMDB08762	C00157<br>C00157<br>C00157<br>C00157<br>C00157<br>C00157<br>C00157<br>C00157<br>C00157<br>C00157	Glycerophospholipids<br>Glycerophospholipids<br>Glycerophospholipids<br>Glycerophospholipids<br>Glycerophospholipids<br>Glycerophospholipids<br>Glycerophospholipids<br>Glycerophospholipids<br>Glycerophospholipids<br>Glycerophospholipids
PC aa C42:2	PC aa C42:2	1-Erucoyl-2-eicosenoy	HMDB08570		Glycerophospholipids
PC aa C42:4	PC aa C42:4	1-Erucoyl-2-meadoyl-	HMDB08572		Glycerophospholipids

		docosapentaenoyl-sn-glycero-3-phosphocholine, GPCho(20:0/22:5), GPCho(20:0/22:5n3), GPCho(20:0/22:5w3), GPCho(42:5), Lecithin, PC aa C42:5, PC(20:0/22:5), PC(20:0/22:5n3), PC(20:0/22:5w3), PC(42:5), Phosphatidylcholine(20:0/22:5), Phosphatidylcholine(20:0/22:5n3), Phosphatidylcholine(20:0/22:5w3), Phosphatidylcholine(42:5)			
	PC(20:0/22:5(7Z,10Z,13Z,16Z,19Z))	1-Arachidonyl-2-docosapentaenoyl-sn-glycero-3-phosphocholine, GPCho(20:0/22:5), GPCho(20:0/22:5n3), GPCho(20:0/22:5w3), GPCho(42:5), Lecithin, PC aa C42:5,	HMDB08287	C00157	Glycerophospholipids
PC aa C42:5	PC(20:0/22:5(7Z,10Z,13Z,16Z,19Z))	PC(20:0/22:5),	HMDB08287	C00157	Glycerophospholipids

		docosahexaenoyl-sn-glycero-3-phosphocholine, GPCho(20:0/22:6), GPCho(20:0/22:6n3), GPCho(20:0/22:6w3), GPCho(42:6), Lecithin, PC aa C42:6, PC(20:0/22:6), PC(20:0/22:6n3), PC(20:0/22:6w3), PC(42:6), Phosphatidylcholine(20:0/22:6), Phosphatidylcholine(20:0/22:6n3), Phosphatidylcholine(20:0/22:6w3), Phosphatidylcholine(42:6)			
	PC(20:0/22:6(4Z,7Z,10Z,13Z,16Z,19Z))	1-Arachidonyl-2-docosahexaenoyl-sn-glycero-3-phosphocholine, GPCho(20:0/22:6), GPCho(20:0/22:6n3), GPCho(20:0/22:6w3), GPCho(42:6), Lecithin, PC aa C42:6,	HMDB08288	C00157	Glycerophospholipids
PC aa C42:6	PC(20:0/22:6(4Z,7Z,10Z,13Z,16Z,19Z))	PC(20:0/22:6),	HMDB08288	C00157	Glycerophospholipids

PC ae C30:0	PC(o-14:0/16:0) PC(o-14:0/16:0)	1-Myristyl-2-palmitoyl-sn-glycero-3-phosphocholine, GPCho(14:0/16:0), GPCho(30:0), Lecithin, PC ae C30:0, PC(14:0/16:0), PC(30:0), PC(O-30:0), Phosphatidylcholine(14:0/16:0), Phosphatidylcholine(30:0) 1-Myristyl-2-palmitoyl-sn-glycero-3-phosphocholine, GPCho(14:0/16:0), GPCho(30:0), Lecithin, PC ae C30:0, PC(14:0/16:0), PC(30:0), PC(O-30:0), Phosphatidylcholine(14:0/16:0), Phosphatidylcholine(30:0)	HMDB13341 HMDB13341		Glycerophospholipids Glycerophospholipids

PC ae C30:1	PC(o-14:0/16:1(9Z)) PC(o-14:0/16:1(9Z))	palmitoleoyl-sn-glycero-3-phosphocholine, GPCho(14:0/16:1), GPCho(14:0/16:1n7), GPCho(14:0/16:1w7), GPCho(30:1), Lecithin, PC ae C30:1, PC(14:0/16:1), PC(14:0/16:1n7), PC(14:0/16:1w7), PC(30:1), PC(O-30:1), Phosphatidylcholine(14:0/16:1), Phosphatidylcholine(14:0/16:1n7), Phosphatidylcholine(14:0/16:1w7), Phosphatidylcholine(30:1) 1-Myristyl-2-palmitoleoyl-sn-glycero-3-phosphocholine, GPCho(14:0/16:1), GPCho(14:0/16:1n7), GPCho(14:0/16:1w7), GPCho(30:1), Lecithin, PC ae C30:1,	HMDB13402 HMDB13402		Glycerophospholipids Glycerophospholipids
PC ae C30:2	PC ae C30:2				
PC ae C32:1	PC ae C32:1				
PC ae C32:2	PC ae C32:2				

PC ae C34:0	PC(o-16:0/18:0)<br>PC(o-16:0/18:0)	1-Palmityl-2-stearoyl-sn-glycero-3-phosphocholine, GPCho(16:0/18:0), GPCho(34:0), Lecithin, PC ae C34:0, PC(16:0/18:0), PC(34:0), PC(O-34:0), Phosphatidylcholine(16:0/18:0), Phosphatidylcholine(34:0)<br>1-Palmityl-2-stearoyl-sn-glycero-3-phosphocholine, GPCho(16:0/18:0), GPCho(34:0), Lecithin, PC ae C34:0, PC(16:0/18:0), PC(34:0), PC(O-34:0), Phosphatidylcholine(16:0/18:0), Phosphatidylcholine(34:0)	HMDB13405<br>HMDB13405		Glycerophospholipids<br>Glycerophospholipids
PC ae C34:1	PC ae C34:1				

PC ae C34:2	PC(O-16:0/18:2(9Z,12Z)) PC(O-16:0/18:2(9Z,12Z))	12Z-octadecadienoyl)-sn-glycero-3-phosphocholine, 2-(9Z, 12Z-Octadecadienoyl)-1-hexadecyl-sn-glycero-3-phosphocholine, PC ae C34:2, PC(16:0e/18:2(9Z, 12Z)), PC(O-34:2), [(2R)-3-hexadecoxy-2-[(9Z, 12Z)-octadeca-9, 12-dienoyl]oxypropyl] 2-trimethylazaniumylethyl phosphate 1-Hexadecyl-2-(9Z, 12Z-octadecadienoyl)-sn-glycero-3-phosphocholine, 2-(9Z, 12Z-Octadecadienoyl)-1-hexadecyl-sn-glycero-3-phosphocholine, PC ae C34:2, PC(16:0e/18:2(9Z, 12Z)), PC(O-34:2), [(2R)-3-hexadecoxy-2-[(9Z, 12Z)-octadeca-9, 12-	HMDB11151 HMDB11151		Glycerophospholipids Glycerophospholipids

PC ae C34:3	PC(P-16:0/18:2(9Z,12Z))<br>PC(P-16:0/18:2(9Z,12Z))	linoleoyl-sn-glycero-3-phosphocholine, GPCho(16:0/18:2), GPCho(16:0/18:2n6), GPCho(16:0/18:2w6), GPCho(34:2), Lecithin, PC aa C34:2, PC(16:0/18:2), PC(16:0/18:2n6), PC(16:0/18:2w6), PC(34:2), Phosphatidylcholine(16:0/18:2), Phosphatidylcholine(16:0/18:2n6), Phosphatidylcholine(16:0/18:2w6), Phosphatidylcholine(34:2)<br>1-(1-Enyl-palmitoyl)-2-linoleoyl-sn-glycero-3-phosphocholine, GPCho(16:0/18:2), GPCho(16:0/18:2n6), GPCho(16:0/18:2w6), GPCho(34:2), Lecithin, PC aa C34:2, PC(16:0/18:2), PC(16:0/18:2n6), PC(16:0/18:2w6),	HMDB11211<br>HMDB11211		Glycerophospholipids<br>Glycerophospholipids

		arachidonyl-sn-glycero-3-phosphocholine, GPCho(16:0/20:0), GPCho(36:0), Lecithin, PC ae C36:0, PC(16:0/20:0), PC(36:0), PC(O-36:0), Phosphatidylcholine(16:0/20:0), Phosphatidylcholine(36:0) 1-Stearyl-2-stearoyl-sn-glycero-3-phosphocholine, GPCho(18:0/18:0), GPCho(36:0), Lecithin, PC ae C36:0, PC(18:0/18:0), PC(36:0), PC(O-36:0), Phosphatidylcholine(18:0/18:0), Phosphatidylcholine(36:0)			
PC ae C36:0	PC(o-16:0/20:0) PC(o-18:0/18:0) PC(o-16:0/20:0) PC(o-18:0/18:0)	1-Palmityl-2-arachidonyl-sn-glycero-3-phosphocholine,	HMDB13406 HMDB13417 HMDB13406 HMDB13417		Glycerophospholipids Glycerophospholipids Glycerophospholipids Glycerophospholipids
PC ae C36:1	PC ae C36:1	1-Oleyl-2-stearoyl-sn-(	HMDB13427		Glycerophospholipids

PC ae C36:2	PC(P-18:0/18:1(9Z))<br>PC(P-18:0/18:1(9Z))	oleoyl-sn-glycero-3-phosphocholine, GPCho(18:0/18:1), GPCho(18:0/18:1n9), GPCho(18:0/18:1w9), GPCho(36:1), Lecithin, PC aa C36:1, PC(18:0/18:1), PC(18:0/18:1n9), PC(18:0/18:1w9), PC(36:1), Phosphatidylcholine(18:0/18:1), Phosphatidylcholine(18:0/18:1n9), Phosphatidylcholine(18:0/18:1w9), Phosphatidylcholine(36:1)<br>1-(1-Enyl-stearoyl)-2-oleoyl-sn-glycero-3-phosphocholine, GPCho(18:0/18:1), GPCho(18:0/18:1n9), GPCho(18:0/18:1w9), GPCho(36:1), Lecithin, PC aa C36:1, PC(18:0/18:1), PC(18:0/18:1n9), PC(18:0/18:1w9),	HMDB11243<br>HMDB11243		Glycerophospholipids<br>Glycerophospholipids
PC ae C36:3	PC ae C36:3	1-Oleyl-2-linoleoyl-sn-	HMDB13429		Glycerophospholipids
PC ae C36:4	PC ae C36:4	1-Linoleyl 2-linoleoyl-s	HMDB13435		Glycerophospholipids

PC ae C36:5	PC(P-16:0/20:4(5Z,8Z,11Z,14Z))<br>PC(P-16:0/20:4(5Z,8Z,11Z,14Z))	Phosphatidylcholine(dm16:0/20:4(5Z, 8Z, 11Z, 14Z))<br>Phosphatidylcholine(dm16:0/20:4(5Z, 8Z, 11Z, 14Z))	HMDB11220<br>HMDB11220		Glycerophospholipids<br>Glycerophospholipids

PC ae C38:0	PC(o-16:0/22:0)<br>PC(o-18:0/20:0)<br>PC(o-16:0/22:0)<br>PC(o-18:0/20:0)	behenoyl-sn-glycero-<br>3-phosphocholine,<br>GPCho(16:0/22:0),<br>GPCho(38:0),<br>Lecithin, PC ae<br>C38:0,<br>PC(16:0/22:0),<br>PC(38:0), PC(O-<br>38:0),<br>Phosphatidylcholine(<br>16:0/22:0),<br>Phosphatidylcholine(<br>38:0)<br>1-Stearyl-2-<br>arachidonyl-sn-<br>glycero-3-<br>phosphocholine,<br>GPCho(18:0/20:0),<br>GPCho(38:0),<br>Lecithin, PC ae<br>C38:0,<br>PC(18:0/20:0),<br>PC(38:0), PC(O-<br>38:0),<br>Phosphatidylcholine(<br>18:0/20:0),<br>Phosphatidylcholine(<br>38:0)<br>1-Palmityl-2-<br>behenoyl-sn-glycero-<br>3-phosphocholine,<br>GPCho(16:0/22:0),	HMDB13408<br>HMDB13419<br>HMDB13408<br>HMDB13419		Glycerophospholipids<br>Glycerophospholipids<br>Glycerophospholipids<br>Glycerophospholipids
PC ae C38:1	PC ae C38:1	1-Oleyl-2-arachidonyl-	HMDB13430		Glycerophospholipids
PC ae C38:2	PC ae C38:2	1-Oleyl-2-eicosenoyl-s	HMDB13431		Glycerophospholipids
PC ae C38:3	PC ae C38:3	1-Arachidyl-2-g-linoler	HMDB13439		Glycerophospholipids

PC ae C38:4	PC(o-18:0/20:4(8Z,11Z,14Z,17Z))<br>PC(o-18:0/20:4(8Z,11Z,14Z,17Z))	1-Stearyl-2-eicsoatetraenoyl-sn-glycero-3-phosphocholine, 1-Stearyl-2-eicsoic acid, GPCho(18:0/20:4), GPCho(18:0/20:4n3), GPCho(18:0/20:4w3), GPCho(38:4), Lecithin, PC ae C38:4, PC(18:0/20:4), PC(18:0/20:4n3), PC(18:0/20:4w3), PC(38:4), PC(O-38:4), Phosphatidylcholine(18:0/20:4), Phosphatidylcholine(18:0/20:4n3), Phosphatidylcholine(18:0/20:4w3), Phosphatidylcholine(38:4)<br>1-Stearyl-2-eicsoate, 1-Stearyl-2-eicsoatetraenoyl-sn-glycero-3-phosphocholine, 1-Stearyl-2-eicsoic acid,	HMDB13420<br>HMDB13420		Glycerophospholipids<br>Glycerophospholipids

		Oleyl-2-eicsoatetraenoyl-sn-glycero-3-phosphocholine, 1-Oleyl-2-eicsoic acid, GPCho(18:1/20:4), GPCho(18:1n9/20:4n3), GPCho(18:1w9/20:4w3), GPCho(38:5), Lecithin, PC ae C38:5, PC(18:1/20:4), PC(18:1n9/20:4n3), PC(18:1w9/20:4w3), PC(38:5), PC(O-38:5), Phosphatidylcholine(18:1/20:4), Phosphatidylcholine(18:1n9/20:4n3), Phosphatidylcholine(18:1w9/20:4w3), Phosphatidylcholine(38:5)			
PC ae C38:5	PC(o-18:1(9Z)/20:4(8Z,11Z,14Z,17Z))<br>PC(o-18:1(9Z)/20:4(8Z,11Z,14Z,17Z))	1-Oleyl-2-eicsoate, 1-Oleyl-2-eicsoatetraenoyl-sn-glycero-3-phosphocholine, 1-Oleyl-2-eicsoic acid, GPCho(18:1/20:4),	HMDB13432<br>HMDB13432		Glycerophospholipids<br>Glycerophospholipids

		docosahexaenoyl-sn-glycero-3-phosphocholine, GPCho(16:0/22:6), GPCho(16:0/22:6n3), GPCho(16:0/22:6w3), GPCho(38:6), Lecithin, PC ae C38:6, PC(16:0/22:6), PC(16:0/22:6n3), PC(16:0/22:6w3), PC(38:6), PC(O-38:6), Phosphatidylcholine(16:0/22:6), Phosphatidylcholine(16:0/22:6n3), Phosphatidylcholine(16:0/22:6w3), Phosphatidylcholine(38:6)			
	PC(o-16:0/22:6(4Z,7Z,10Z,13Z,16Z,19Z))	1-Palmityl-2-docosahexaenoyl-sn-glycero-3-phosphocholine, GPCho(16:0/22:6), GPCho(16:0/22:6n3), GPCho(16:0/22:6w3), GPCho(38:6), Lecithin, PC ae	HMDB13409		Glycerophospholipids
PC ae C38:6	PC(o-16:0/22:6(4Z,7Z,10Z,13Z,16Z,19Z))	C38:6,	HMDB13409		Glycerophospholipids

PC ae C40:0	PC(o-18:0/22:0) PC(o-18:0/22:0)	1-Stearyl-2-behenoyl-sn-glycero-3-phosphocholine, GPCho(18:0/22:0), GPCho(40:0), Lecithin, PC ae C40:0, PC(18:0/22:0), PC(40:0), PC(O-40:0), Phosphatidylcholine(18:0/22:0), Phosphatidylcholine(40:0) 1-Stearyl-2-behenoyl-sn-glycero-3-phosphocholine, GPCho(18:0/22:0), GPCho(40:0), Lecithin, PC ae C40:0, PC(18:0/22:0), PC(40:0), PC(O-40:0), Phosphatidylcholine(18:0/22:0), Phosphatidylcholine(40:0)	HMDB13421 HMDB13421		Glycerophospholipids Glycerophospholipids
PC ae C40:1	PC ae C40:1	1-Oleyl-2-behenoyl-sn	HMDB13433		Glycerophospholipids
PC ae C40:2	PC ae C40:2	1-Linoleyl-2-behenoyl-	HMDB13437		Glycerophospholipids
PC ae C40:3	PC ae C40:3	1-Behenyl-2-g-linolen	HMDB13445		Glycerophospholipids
PC ae C40:4	PC ae C40:4	1-Arachidyl-2-eicsoate	HMDB13442		Glycerophospholipids
PC ae C40:5	PC ae C40:5	1-Eicosenyl-2-eicsoat	HMDB13444		Glycerophospholipids

PC ae C40:6	PC(o-18:0/22:6(4Z,7Z,10Z,13Z,16Z,19Z))<br>PC(o-18:0/22:6(4Z,7Z,10Z,13Z,16Z,19Z))	docosahexaenoyl-sn-glycero-3-phosphocholine, GPCho(18:0/22:6), GPCho(18:0/22:6n3), GPCho(18:0/22:6w3), GPCho(40:6), Lecithin, PC ae C40:6, PC(18:0/22:6), PC(18:0/22:6n3), PC(18:0/22:6w3), PC(40:6), PC(O-40:6), Phosphatidylcholine(18:0/22:6), Phosphatidylcholine(18:0/22:6n3), Phosphatidylcholine(18:0/22:6w3), Phosphatidylcholine(40:6)<br>1-Stearyl-2-docosahexaenoyl-sn-glycero-3-phosphocholine, GPCho(18:0/22:6), GPCho(18:0/22:6n3), GPCho(18:0/22:6w3), GPCho(40:6), Lecithin, PC ae C40:6,	HMDB13422<br>HMDB13422		Glycerophospholipids<br>Glycerophospholipids

PC ae C42:0	PC(o-20:0/22:0) PC(o-20:0/22:0)	1-Arachidyl-2-behenoyl-sn-glycero-3-phosphocholine, GPCho(20:0/22:0), GPCho(42:0), Lecithin, PC ae C42:0, PC(20:0/22:0), PC(42:0), PC(O-42:0), Phosphatidylcholine(20:0/22:0), Phosphatidylcholine(42:0) 1-Arachidyl-2-behenoyl-sn-glycero-3-phosphocholine, GPCho(20:0/22:0), GPCho(42:0), Lecithin, PC ae C42:0, PC(20:0/22:0), PC(42:0), PC(O-42:0), Phosphatidylcholine(20:0/22:0), Phosphatidylcholine(42:0)	HMDB13443 HMDB13443		Glycerophospholipids Glycerophospholipids
PC ae C42:1	PC ae C42:1	1-Oleyl-2-lignoceroyl-s	HMDB13434		Glycerophospholipids
PC ae C42:2	PC ae C42:2	1-Linoleyl-2-lignocero	HMDB13438		Glycerophospholipids
PC ae C42:3	PC ae C42:3	1-Lignoceryl-2-a-linole	HMDB13459		Glycerophospholipids
PC ae C42:4	PC ae C42:4	1-Behenyl-2-eicsoate,	HMDB13448		Glycerophospholipids
PC ae C42:5	PC ae C42:5	1-Erucyl-2-eicsoate, 1	HMDB13451		Glycerophospholipids
PC ae C44:3	PC ae C44:3	1-Behenyl-2-(10Z, 13Z	HMDB13449		Glycerophospholipids
PC ae C44:4	PC ae C44:4	1-Erucyl-2-(10Z, 13Z,	HMDB13453		Glycerophospholipids

PC ae C44:5	PC ae C44:5	1-Docosadienyl-2-(10	HMDB13456		Glycerophospholipids
PC ae C44:6	PC ae C44:6	1-Docosatrienyl-2-(10	HMDB13457		Glycerophospholipids
PEA	1-Phenylethylamine<br>2-Phenylethylamine<br>1-Phenylethylamine<br>2-Phenylethylamine	Aminoethyl)benzene, 1-Amino-1-phenylethane, 1-Fenylethylamin, 1-Phenyl-1-ethanamine, 1-Phenylethanamine, 1-Phenylethylamine, a-Aminoethylbenzene, a-Methylbenzenemethanamine, a-Methylbenzylamine, a-Phenethylamine, a-Phenylethylamine, alpha-Aminoethylbenzene, alpha-Methylbenzenemethanamine, alpha-Methylbenzylamine, alpha-Phenethylamine, alpha-Phenylethylamine, Sumine 2079<br>(2-Aminoethyl)benzene, (2-Aminoethyl)polystyrene, 1-Amino-2-phenylethane, 1-	HMDB02017<br>HMDB12275<br>HMDB02017<br>HMDB12275	C02455<br>C05332<br>C02455<br>C05332	Phenylmethylamines<br>Phenethylamines<br>Phenylmethylamines<br>Phenethylamines

	L-Phenylalanine	Phenylalanine, (L)-Phenylalanine, (S)-(-)-Phenylalanine, (S)-2-amino-3-phenylpropanoate, (S)-2-amino-3-phenylpropanoic acid, (S)-2-Amino-3-phenylpropionate, (S)-2-Amino-3-phenylpropionic acid, (S)-alpha-Amino-benzenepropanoate, (S)-alpha-Amino-benzenepropanoic acid, (S)-alpha-Amino-beta-phenylpropionate, (S)-alpha-Amino-beta-phenylpropionic acid, (S)-alpha-Aminobenzenepropanoate, (S)-alpha-Aminobenzenepropanoic acid, (S)-alpha-Aminohydrocinnamate, (S)-alpha-Aminohydrocinnamic acid, (S)-Phenylalanine, 3-Phenyl-L-alanine,	HMDB00159	C00079	Amino Acids and Derivatives
Phe	L-Phenylalanine	alpha-	HMDB00159	C00079	Amino Acids and Derivatives

	L-Proline	Pyrrolidinecarboxylate, (-)-2-Pyrrolidinecarboxylic acid, (-)-Proline, (S)-(-)-Proline, (S)-(-)-Pyrrolidine-2-carboxylate, (S)-(-)-Pyrrolidine-2-carboxylic acid, (S)-2-Carboxypyrrolidine, (S)-2-Pyrralidinecarboxylate, (S)-2-Pyrralidinecarboxylic acid, (S)-2-Pyrrolidinecarboxylate, (S)-2-Pyrrolidinecarboxylic acid, (S)-Proline, 2-Pyrrolidinecarboxylate, 2-Pyrrolidinecarboxylic acid, Proline (2R)-pyrrolidine-2-carboxylate, (2R)-pyrrolidine-2-carboxylic acid, (R)-pyrrolidine-2-carboxylate, (R)-	HMDB00162	C00148	Amino Acids and Derivatives
	D-Proline	pyrrolidine-2-	HMDB03411	C00763	Amino Acids and Derivatives
	L-Proline	carboxylic acid, D-	HMDB00162	C00148	Amino Acids and Derivatives
Pro	D-Proline	Prolin, D-Proline, R)-	HMDB03411	C00763	Amino Acids and Derivatives

	Putrescine	1, 4-Butylenediamine, 1, 4-Diaminobutane, 1, 4-Tetramethylenediamine, Butylenediamine, Putrescin, Tetramethyldiamine, Tetramethylenediamine	HMDB01414	C00134	Alkylamines
	Putrescine	1, 4-Butanediamine, 1, 4-Butylenediamine, 1, 4-Diaminobutane, 1, 4-Tetramethylenediamine, Butylenediamine, Putrescin, Tetramethyldiamine, Tetramethylenediamine	HMDB01414	C00134	Alkylamines
Putrescine	Putrescine	1, 4-Butanediamine, 1, 4-Butylenediamine, 1, 4-Diaminobutane, 1, 4-Tetramethylenediamine, Butylenediamine, Putrescin, Tetramethyldiamine, Tetramethylenediamine	HMDB01414	C00134	Alkylamines

SDMA	Symmetric dimethylarginine Symmetric dimethylarginine	dimethylarginine, N(G1), N(G2)-Dimethylarginine, N, N'-Dimethylarginine, N5-((methylamino)(methylimino)methyl)-L-Ornithine, N5-(N, N'-dimethylamidino)-L-Ornithine, N5-[bis(methylamino)methylene]-L-Ornithine, NG, N'G-Dimethyl-L-arginine, NG, N'G-dimethylarginine, NG, NG'-dimethylarginine, SDMA Guanidino-N(1), N(2)-dimethylarginine, N(G1), N(G2)-Dimethylarginine, N, N'-Dimethylarginine, N5-((methylamino)(methylimino)methyl)-L-Ornithine, N5-(N, N'-dimethylamidino)-L-Ornithine, N5-[bis(methylamino)methylene]-L-Ornithine, NG, N'G-Dimethyl-L-	HMDB03334 HMDB03334		Amino Acids and Derivatives Amino Acids and Derivatives
SM (OH) C14:1	SM (OH) C14:1				
SM (OH) C16:1	SM (OH) C16:1				
SM (OH) C22:1	SM (OH) C22:1				

SM (OH) C22:2	SM (OH) C22:2				
SM (OH) C24:1	SM (OH) C24:1				
SM C16:0	SM C16:0	C16 Sphingomyelin, N	HMDB10169		Sphingolipids
SM C16:1	Sphingomyelin with ac		HMDB29216		Sphingolipids
SM C18:0	SM C18:0	N-(Octadecanoyl)-sph	HMDB01348		Sphingolipids
SM C18:1	C18:1;11Z-enoyl Sphingomyelin C18:1;9Z-enoyl Sphingomyelin	N-(11Z-Octadecenoyl)-sphing-4-enine-1-phosphocholine, SPH(d18:1/18:1(11Z)), Sphingomyelin, Sphingomyelin (d18:1/18:1(11Z)) C18:1 Sphingomyelin, N-(9Z-Octadecenoyl)-sphing-4-enine-1-phosphocholine, SPH(d18:1/18:1(11Z)), Sphingomyelin, Sphingomyelin (d18:1/18:1(11Z))	HMDB12100 HMDB12101	C00550 C00550	Sphingolipids Sphingolipids
SM C20:2	SM C20:2				
SM C22:3	SM C22:3				
SM C24:0	SM C24:0				
SM C24:1	SM(d18:1/24:1(15Z)) SM(d18:1/24:1(15Z))	C24:1 Sphingomyelin, N-(15Z-Tetracosenoyl)-sphing-4-enine-1-phosphocholine C24:1 Sphingomyelin, N-(15Z-Tetracosenoyl)-sphing-4-enine-1-phosphocholine	HMDB12107 HMDB12107	C00550 C00550	Sphingolipids Sphingolipids

SM C26:0	SM C26:0	N-(Hexacosanoyl)-sph	HMDB11698		Sphingolipids
	SM(d18:0/26:1(17Z))	C26:1 Sphingomyelin, N-(Tricosanoyl)-sphing-4-enine-1-phosphocholine,	HMDB13461	C00550	Sphingolipids
SM C26:1	SM(d18:0/26:1(17Z))	Sphingomyelin C26:1 Sphingomyelin, N-(Tricosanoyl)-sphing-4-enine-1-phosphocholine, Sphingomyelin	HMDB13461	C00550	Sphingolipids

		amino-3-hydroxy-Propanoate, (S)-2-amino-3-hydroxy-Propanoic acid, (S)-2-Amino-3-hydroxypropanoate, (S)-2-Amino-3-hydroxypropanoic acid, (S)-a-Amino-b-hydroxypropionate, (S)-a-Amino-b-hydroxypropionic acid, (S)-alpha-Amino-beta-hydroxypropionate, (S)-alpha-Amino-beta-hydroxypropionic acid, (S)-b-Amino-3-hydroxypropionate, (S)-b-Amino-3-hydroxypropionic acid, (S)-beta-Amino-3-hydroxypropionate, (S)-beta-Amino-3-hydroxypropionic acid, (S)-Serine, 2-Amino-3-			
	L-Serine	hydroxypropanoate,	HMDB00187	C00065	Amino Acids and Derivatives
	D-Serine	2-Amino-3-	HMDB03406	C00740	Amino Acids and Derivatives
	L-Serine	hydroxypropanoic	HMDB00187	C00065	Amino Acids and Derivatives
Ser	D-Serine	acid, 3-Hydroxy-L-	HMDB03406	C00740	Amino Acids and Derivatives

		indol-5-ol, 3-(2-Aminoethyl)indol-5-ol, 3-(b-Aminoethyl)-5-hydroxyindole, 5-HT, 5-HTA, 5-Hydroxy-3-(b-aminoethyl)indole, 5-Hydroxy-tryptamine, 5-Hydroxyltryptamine, 5-Hydroxytriptamine, 5-Hydroxytryptamine, Antemovis, DS substance, Enteramin, Enteramine			
	Serotonin	3-(2-Aminoethyl)-1H-indol-5-ol, 3-(2-Aminoethyl)indol-5-ol, 3-(b-Aminoethyl)-5-hydroxyindole, 5-HT, 5-HTA, 5-Hydroxy-3-(b-aminoethyl)indole, 5-Hydroxy-tryptamine, 5-Hydroxyltryptamine, 5-Hydroxytriptamine, 5-Hydroxytryptamine, Antemovis, DS	HMDB00259	C00780	Indoles
	Serotonin	substance,	HMDB00259	C00780	Indoles
Serotonin	Serotonin	Enteramin,	HMDB00259	C00780	Indoles

Spermidine	Spermidine<br>Spermidine<br>Spermidine	Triazadecane, 1, 8-Diamino-4-azaoctane, 4-Azaoctamethylenediamine, 4-Azaoctane-1, 8-diamine, Aminopropylbutandiamine, N-(3-Aminopropyl)-1, 4-butane-diamine, N-(3-Aminopropyl)-1, 4-butanediamine, N-(3-Aminopropyl)-1, 4-diamino-butane, N-(3-Aminopropyl)-1, 4-diaminobutane, N-(3-Aminopropyl)-4-aminobutylamine, N-(4-Aminobutyl)-1, 3-diaminopropane, N-(gamma-Aminopropyl)tetramethylenediamine, SPD, Spermidin, Spermidine<br>1, 5, 10-Triazadecane, 1, 8-Diamino-4-azaoctane, 4-Azaoctamethylenediamine, 4-Azaoctane-1, 8-diamine,	HMDB01257<br>HMDB01257<br>HMDB01257	C00315<br>C00315<br>C00315	Alkylamines<br>Alkylamines<br>Alkylamines

		Tetraazatetradecane, 4, 9-Diaza-1, 12-dodecanediamine, 4, 9-Diazadodecamethylenediamine, 4, 9-Diazadodecane-1, 12-diamine, Diaminopropyl-tetramethylenediamine, Diaminopropyltetramethylenediamine, Gerontine, Musculamine, N, N'-Bis(3-aminopropyl)-1, 4-Butanediamine, N, N'-Bis(3-aminopropyl)-1, 4-tetramethylenediamine, N, N'-Bis(3-aminopropyl)butane-1, 4-diamine, N1, N4-bis(3-aminopropyl)-1, 4-butanediamine, Neuridine, Spermin, Spermine, Spermine dihydrate, Spermine puriss, SPM			
	Spermine	1, 5, 10, 14-	HMDB01256	C00750	Alkylamines
	Spermine	Tetraazatetradecane,	HMDB01256	C00750	Alkylamines
Spermine	Spermine	4, 9-Diaza-1, 12-	HMDB01256	C00750	Alkylamines

		sulfonate, 1-Aminoethane-2-sulfonic acid, 2-Aminoethanesulfonate, 2-Aminoethanesulfonic acid, 2-Aminoethylsulfonate, 2-Aminoethylsulfonic acid, 2-Sulfoethylamine, Aminoethylsulfonate, Aminoethylsulfonic acid, b-Aminoethylsulfonate, b-Aminoethylsulfonic acid, beta-Aminoethylsulfonate, beta-Aminoethylsulfonic acid, Taurine 1-Aminoethane-2-sulfonate, 1-Aminoethane-2-sulfonic acid, 2-Aminoethanesulfonate, 2-Aminoethanesulfonic acid, 2-			
	Taurine	Aminoethylsulfonate,	HMDB00251	C00245	Sulfonic Acids and Derivatives
	Taurine	2-Aminoethylsulfonic	HMDB00251	C00245	Sulfonic Acids and Derivatives
Taurine	Taurine	acid, 2-	HMDB00251	C00245	Sulfonic Acids and Derivatives

	L-Threonine	Threonine, (2S, 3R)-2-Amino-3-hydroxybutyrate, (2S, 3R)-2-Amino-3-hydroxybutyric acid, (R-(R*, S*))-2-Amino-3-hydroxybutanoate, (R-(R*, S*))-2-Amino-3-hydroxybutanoic acid, (S)-Threonine, 2-Amino-3-hydroxybutanoate, 2-Amino-3-hydroxybutanoic acid, 2-Amino-3-hydroxybutyrate, 2-Amino-3-hydroxybutyric acid, L-(-)-Threonine, L-2-Amino-3-hydroxybutyrate, L-2-Amino-3-hydroxybutyric acid, L-alpha-Amino-beta-hydroxybutyrate, L-alpha-Amino-beta-hydroxybutyric acid, Threonin, Threonine, [R-(R*, S*)]-2-amino-	HMDB00167	C00188	Amino Acids and Derivatives
	L-Allothreonine	3-hydroxy-Butanoate,	HMDB04041	C05519	Amino Acids and Derivatives
	L-Threonine	[R-(R*, S*)]-2-amino-	HMDB00167	C00188	Amino Acids and Derivatives
Thr	L-Allothreonine	3-hydroxy-Butanoic	HMDB04041	C05519	Amino Acids and Derivatives

Supplement table 1

	L-Tryptophan		HMDB00929	C00078	Amino Acids and Derivatives
	D-Tryptophan		HMDB13609	C00525	Amino Acids and Derivatives
	L-Tryptophan		HMDB00929	C00078	Amino Acids and Derivatives
Trp	D-Tryptophan	(-)-tryptophan, (2S)-2-	HMDB13609	C00525	Amino Acids and Derivatives

Tyr	L-Tyrosine<br>L-Tyrosine	hydroxyhydrocinnamate, (-)-a-Amino-p-hydroxyhydrocinnamic acid, (-)-alpha-Amino-p-hydroxyhydrocinnamate, (-)-alpha-Amino-p-hydroxyhydrocinnamic acid, (S)-(-)-Tyrosine, (S)-2-Amino-3-(p-hydroxyphenyl)propionate, (S)-2-Amino-3-(p-hydroxyphenyl)propionic acid, (S)-3-(p-Hydroxyphenyl)alanine, (S)-a-amino-4-hydroxy-Benzenepropanoate, (S)-a-amino-4-hydroxy-Benzenepropanoic acid, (S)-a-Amino-4-hydroxybenzenepropanoate, (S)-a-Amino-4-hydroxybenzenepropanoic acid, (S)-alpha-amino-4-hydroxy-Benzenepropanoate,	HMDB00158<br>HMDB00158	C00082<br>C00082	Amino Acids and Derivatives<br>Amino Acids and Derivatives

	L-Valine	methylbutanoate, (2S)-2-Amino-3-methylbutanoic acid, (S)-2-amino-3-methyl-Butanoate, (S)-2-amino-3-methyl-Butanoic acid, (S)-2-Amino-3-methylbutanoate, (S)-2-Amino-3-methylbutanoic acid, (S)-2-Amino-3-methylbutyrate, (S)-2-Amino-3-methylbutyric acid, (S)-a-Amino-b-methylbutyrate, (S)-a-Amino-b-methylbutyric acid, (S)-alpha-Amino-beta-methylbutyrate, (S)-alpha-Amino-beta-methylbutyric acid, (S)-Valine, 2-Amino-3-methylbutanoate, 2-Amino-3-methylbutanoic acid, 2-Amino-3-methylbutyrate, 2-Amino-3-	HMDB00883	C00183	Amino Acids and Derivatives
Val	L-Valine	methylbutyric acid, L-	HMDB00883	C00183	Amino Acids and Derivatives

lysoPC a C14:0	LysoPC(14:0)<br>LysoPC(14:0)	1-Myristoyl-glycero-3-phosphocholine, LPC(14:0), LPC(14:0/0:0), LyPC(14:0), LyPC(14:0/0:0), lysoPC a C14:0, LysoPC(14:0), LysoPC(14:0/0:0), Lysophosphatidylcholine(14:0), Lysophosphatidylcholine(14:0/0:0)<br>1-Myristoyl-glycero-3-phosphocholine, LPC(14:0), LPC(14:0/0:0), LyPC(14:0), LyPC(14:0/0:0), lysoPC a C14:0, LysoPC(14:0), LysoPC(14:0/0:0), Lysophosphatidylcholine(14:0), Lysophosphatidylcholine(14:0/0:0)	HMDB10379<br>HMDB10379	C04230<br>C04230	Glycerophospholipids<br>Glycerophospholipids

lysoPC a C16:0	LysoPC(16:0)<br>LysoPC(16:0)	1-Palmitoyl-glycero-3-phosphocholine, 1-Palmitoyl-lysophosphatidylcholine, LPC(16:0), LPC(16:0/0:0), LyPC(16:0), LyPC(16:0/0:0), lysoPC a C16:0, LysoPC(16:0), LysoPC(16:0/0:0), Lysophosphatidylcholine(16:0), Lysophosphatidylcholine(16:0/0:0)<br>1-Palmitoyl-glycero-3-phosphocholine, 1-Palmitoyl-lysophosphatidylcholine, LPC(16:0), LPC(16:0/0:0), LyPC(16:0), LyPC(16:0/0:0), lysoPC a C16:0, LysoPC(16:0), LysoPC(16:0/0:0), Lysophosphatidylcholine(16:0), Lysophosphatidylcholine(16:0/0:0)	HMDB10382<br>HMDB10382	C04230<br>C04230	Glycerophospholipids<br>Glycerophospholipids

lysoPC a C16:1	LysoPC(16:1(9Z))<br>LysoPC(16:1(9Z))	glycero-3-<br>phosphocholine,<br>LPC(16:1),<br>LPC(16:1/0:0),<br>LPC(16:1n7/0:0),<br>LPC(16:1w7/0:0),<br>LyPC(16:1),<br>LyPC(16:1/0:0),<br>LyPC(16:1n7/0:0),<br>LyPC(16:1w7/0:0),<br>lysoPC a C16:1,<br>LysoPC(16:1),<br>LysoPC(16:1/0:0),<br>LysoPC(16:1n7/0:0),<br>LysoPC(16:1w7/0:0),<br>Lysophosphatidylchol<br>ine(16:1),<br>Lysophosphatidylchol<br>ine(16:1/0:0),<br>Lysophosphatidylchol<br>ine(16:1n7/0:0),<br>Lysophosphatidylchol<br>ine(16:1w7/0:0)<br>1-Palmitoleoyl-<br>glycero-3-<br>phosphocholine,<br>LPC(16:1),<br>LPC(16:1/0:0),<br>LPC(16:1n7/0:0),<br>LPC(16:1w7/0:0),<br>LyPC(16:1),<br>LyPC(16:1/0:0),	HMDB10383<br>HMDB10383	C04230<br>C04230	Glycerophospholipids<br>Glycerophospholipids

lysoPC a C17:0	LysoPC(17:0)<br>LysoPC(17:0)	1-Heptadecanoyl-glycero-3-phosphocholine, LPC(17:0), LPC(17:0/0:0), LyPC(17:0), LyPC(17:0/0:0), lysoPC a C17:0, LysoPC(17:0), LysoPC(17:0/0:0), Lysophosphatidylcholine(17:0), Lysophosphatidylcholine(17:0/0:0)<br>1-Heptadecanoyl-glycero-3-phosphocholine, LPC(17:0), LPC(17:0/0:0), LyPC(17:0), LyPC(17:0/0:0), lysoPC a C17:0, LysoPC(17:0), LysoPC(17:0/0:0), Lysophosphatidylcholine(17:0), Lysophosphatidylcholine(17:0/0:0)	HMDB12108<br>HMDB12108	C04230<br>C04230	Glycerophospholipids<br>Glycerophospholipids

lysoPC a C18:0	LysoPC(18:0)<br>LysoPC(0:0/18:0)<br>LysoPC(18:0)<br>LysoPC(0:0/18:0)	phosphocholine,<br>LPC(18:0),<br>LPC(18:0/0:0),<br>LyPC(18:0),<br>LyPC(18:0/0:0),<br>lysoPC a C18:0,<br>LysoPC(18:0),<br>LysoPC(18:0/0:0),<br>Lysophosphatidylcholine(18:0),<br>Lysophosphatidylcholine(18:0/0:0)<br>1-Hydroxy-2-Octadecanoyl-sn-glycero-3-phosphocholine, 1-Hydroxy-2-stearoyl-sn-glycero-3-phosphocholine,<br>Lysophosphatidylcholine, Stearoyl lysophosphatidylcholine<br>1-Stearoyl-glycero-3-phosphocholine,<br>LPC(18:0),<br>LPC(18:0/0:0),<br>LyPC(18:0),<br>LyPC(18:0/0:0),<br>lysoPC a C18:0,<br>LysoPC(18:0),<br>LysoPC(18:0/0:0),	HMDB10384<br>HMDB11128<br>HMDB10384<br>HMDB11128	C04230<br>C04230	Glycerophospholipids<br>Glycerophospholipids<br>Glycerophospholipids<br>Glycerophospholipids

lysoPC a C18:1	LysoPC(18:1(9Z))<br>LysoPC(18:1(11Z))<br>LysoPC(18:1(9Z))<br>LysoPC(18:1(11Z))	sn-glycero-3-phosphocholine, 1-Oleoyl-sn-glycero-3-phosphocholine, 1-Oleoylglycerophosphocholine, 3-Oleoyl-rac-glycerol-1-phosphorylcholine, Choline phosphate (ester) 3-ester with 1-monoolein, Choline phosphate 3-ester with 1-monoolein, GPCho(18:1(9Z)/0:0) [rac], LPC(18:1n9/0:0), LPC(18:1w9/0:0), LyPC(18:1), LyPC(18:1/0:0), LyPC(18:1w9/0:0), lysoPC a C18:1, LysoPC(18:1), LysoPC(18:1n9/0:0), LysoPC(18:1w9/0:0), Lysophosphatidylcholine(18:1), Lysophosphatidylcholine(18:1/0:0), Lysophosphatidylcholine(18:1n9/0:0), Lysophosphatidylcholine(18:1w9/0:0),	HMDB02815<br>HMDB10385<br>HMDB02815<br>HMDB10385	C04230<br>C04230<br>C04230<br>C04230	Glycerophospholipids<br>Glycerophospholipids<br>Glycerophospholipids<br>Glycerophospholipids

lysoPC a C18:2	LysoPC(18:2(9Z,12Z)) LysoPC(18:2(9Z,12Z))	phosphocholine, LPC(18:2), LPC(18:2/0:0), LPC(18:2n6/0:0), LPC(18:2w6/0:0), LyPC(18:2), LyPC(18:2/0:0), LyPC(18:2n6/0:0), LyPC(18:2w6/0:0), lysoPC a C18:2, LysoPC(18:2), LysoPC(18:2/0:0), LysoPC(18:2n6/0:0), LysoPC(18:2w6/0:0), Lysophosphatidylcholine(18:2), Lysophosphatidylcholine(18:2/0:0), Lysophosphatidylcholine(18:2n6/0:0), Lysophosphatidylcholine(18:2w6/0:0) 1-Linoleoyl-glycero-3-phosphocholine, LPC(18:2), LPC(18:2/0:0), LPC(18:2n6/0:0), LPC(18:2w6/0:0), LyPC(18:2), LyPC(18:2/0:0), LyPC(18:2n6/0:0), LyPC(18:2w6/0:0),	HMDB10386 HMDB10386	C04230 C04230	Glycerophospholipids Glycerophospholipids

lysoPC a C20:3	LysoPC(20:3(5Z,8Z,11Z))<br>LysoPC(20:3(8Z,11Z,14Z))<br>LysoPC(20:3(5Z,8Z,11Z))<br>LysoPC(20:3(8Z,11Z,14Z))	phosphocholine, LPC(20:3), LPC(20:3/0:0), LPC(20:3n9/0:0), LPC(20:3w9/0:0), LyPC(20:3), LyPC(20:3/0:0), LyPC(20:3n9/0:0), LyPC(20:3w9/0:0), lysoPC a C20:3, LysoPC(20:3), LysoPC(20:3/0:0), LysoPC(20:3n9/0:0), LysoPC(20:3w9/0:0), Lysophosphatidylcholine(20:3), Lysophosphatidylcholine(20:3/0:0), Lysophosphatidylcholine(20:3n9/0:0), Lysophosphatidylcholine(20:3w9/0:0)<br>1-Homo-g-linolenoyl-glycero-3-phosphocholine, 1-Homo-gamma-linolenoyl-glycero-3-phosphocholine, LPC(20:3), LPC(20:3/0:0), LPC(20:3n6/0:0), LPC(20:3w6/0:0),	HMDB10393<br>HMDB10394<br>HMDB10393<br>HMDB10394	C04230<br>C04230<br>C04230<br>C04230	Glycerophospholipids<br>Glycerophospholipids<br>Glycerophospholipids<br>Glycerophospholipids

lysoPC a C20:4	LysoPC(20:4(5Z,8Z,1<br>1Z,14Z))<br>LysoPC(20:4(8Z,11Z,<br>14Z,17Z))<br>LysoPC(20:4(5Z,8Z,1<br>1Z,14Z))<br>LysoPC(20:4(8Z,11Z,<br>14Z,17Z))	glycero-3-<br>phosphocholine,<br>LPC(20:4),<br>LPC(20:4/0:0),<br>LPC(20:4n6/0:0),<br>LPC(20:4w6/0:0),<br>LyPC(20:4),<br>LyPC(20:4/0:0),<br>LyPC(20:4n6/0:0),<br>LyPC(20:4w6/0:0),<br>lysoPC a C20:4,<br>LysoPC(20:4),<br>LysoPC(20:4/0:0),<br>LysoPC(20:4n6/0:0),<br>LysoPC(20:4w6/0:0),<br>Lysophosphatidylchol<br>ine(20:4),<br>Lysophosphatidylchol<br>ine(20:4/0:0),<br>Lysophosphatidylchol<br>ine(20:4n6/0:0),<br>Lysophosphatidylchol<br>ine(20:4w6/0:0)<br>1-Eicsoate, 1-<br>Eicsoatetraenoyl-<br>glycero-3-<br>phosphocholine, 1-<br>Eicsoic acid,<br>LPC(20:4),<br>LPC(20:4/0:0),<br>LPC(20:4n3/0:0),<br>LPC(20:4w3/0:0),	HMDB10395<br>HMDB10396<br>HMDB10395<br>HMDB10396	C04230<br>C04230<br>C04230<br>C04230	Glycerophospholipids<br>Glycerophospholipids<br>Glycerophospholipids<br>Glycerophospholipids

lysoPC a C24:0	LysoPC(24:0)<br>LysoPC(24:0)	1-Lignoceroyl-glycero-3-phosphocholine, LPC(24:0), LPC(24:0/0:0), LyPC(24:0), LyPC(24:0/0:0), lysoPC a C24:0, LysoPC(24:0), LysoPC(24:0/0:0), Lysophosphatidylcholine(24:0), Lysophosphatidylcholine(24:0/0:0)<br>1-Lignoceroyl-glycero-3-phosphocholine, LPC(24:0), LPC(24:0/0:0), LyPC(24:0), LyPC(24:0/0:0), lysoPC a C24:0, LysoPC(24:0), LysoPC(24:0/0:0), Lysophosphatidylcholine(24:0), Lysophosphatidylcholine(24:0/0:0)	HMDB10405<br>HMDB10405	C04230<br>C04230	Glycerophospholipids<br>Glycerophospholipids
lysoPC a C26:0	lysoPC a C26:0	lysoPC a C26:0	HMDB29205		Glycerophospholipids
lysoPC a C26:1	lysoPC a C26:1	lysoPC a C26:1	HMDB29220		Glycerophospholipids
lysoPC a C28:0	lysoPC a C28:0	lysoPC a C28:0	HMDB29206		Glycerophospholipids
lysoPC a C28:1	lysoPC a C28:1	lysoPC a C28:1	HMDB29221		Glycerophospholipids
